# Implications of Charge and Heteroatom Dopants on the Thermodynamics and Kinetics of Redox Reactions in Keggin-Type Polyoxometalates

**DOI:** 10.1021/acsmaterialsau.4c00136

**Published:** 2024-11-27

**Authors:** Mamta Dagar, Anyesh De, Zhou Lu, Ellen M. Matson, Agnes E. Thorarinsdottir

**Affiliations:** Department of Chemistry, University of Rochester, Rochester, New York 14627, United States

**Keywords:** polyoxometalates, redox reactions, electron transfer, kinetics, thermodynamics, entropy, Born model

## Abstract

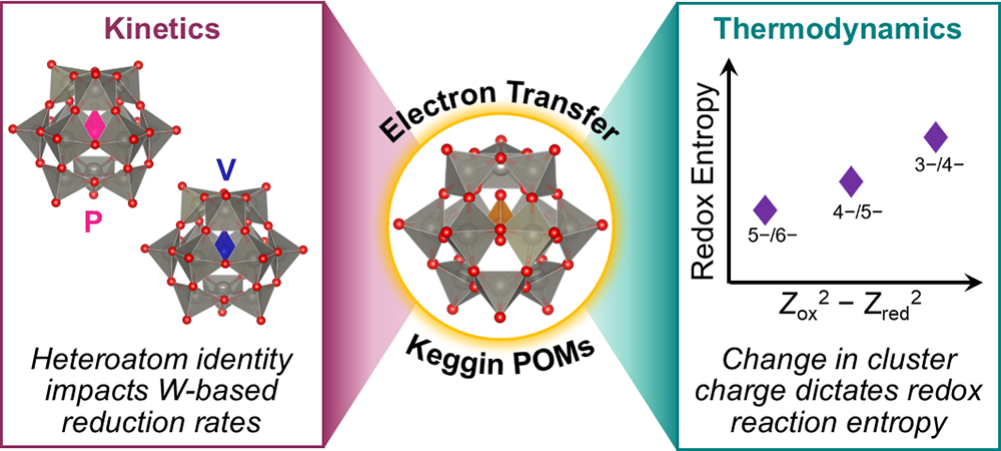

The utilization of polyoxometalate-based materials is largely dictated by their redox properties. Detailed understanding of the thermodynamic and kinetic efficiency of charge transfer is therefore essential to the development of polyoxometalate-based systems for target applications. Toward this end, we report electrochemical studies of a series of heteroatom-doped Keggin-type polyoxotungstate clusters [PW_12_O_40_]^3–^ (**PW**_**12**_), [VW_12_O_40_]^3–^ (**V**_**in**_**W**_**12**_), [P(VW_11_)O_40_]^4–^ (**PV**_**out**_**W**_**11**_), and [V(VW_11_)O_40_]^4–^ (**V**_**in**_**V**_**out**_**W**_**11**_) to elucidate the role of the identity and spatial location of heteroatoms and overall cluster charge on the rate constants of electron transfer and redox reaction entropies. Electrochemical analyses of the polyoxotungstates reveal that the kinetics of electron transfer for W-based redox processes change as a function of the redox activity of the heteroatom, whereas the spatial location of the heteroatom dopant does not significantly impact the electrokinetics. Variable temperature cyclic voltammetry measurements in organic solutions containing noncoordinating electrolyte ions establish that redox reaction entropies are primarily dictated by the overall charge of the clusters. Specifically, the redox entropy exhibits a good linear relationship with the dielectric continuum function *Z*_ox_^2^ – *Z*_red_^2^ (*Z*_ox_ = charge of oxidized species, *Z*_red_ = charge of reduced species). Finally, our experimental data do not show a prominent correlation between the kinetics of electron transfer and redox entropy, implying that the charge-transfer kinetics are not solely governed by structural reorganization. Taken together, these results highlight how structural and electronic parameters can influence the kinetics and thermodynamics of charge transfer in polyoxotungstates and provide insights into the design of polyoxometalate compounds with target redox properties.

Polyoxometalates (POMs) represent an attractive class of redox-active inorganic compounds with unique properties that have led to their use in applications pertaining to medicine, catalysis, energy storage and conversion, electrophotography, and gas sorption.^1^ POMs are composed of multiple transition metal ions, most often tungsten, molybdenum, or vanadium, linked together by bridging oxygen atoms to form three-dimensional structures with large, negative ionic charges. Their redox properties are dictated by structural changes, i.e., arrangement of metal atoms and overall molecular geometry, within the cluster core. For example, one subset of POMs are the Keggin-type clusters, with the general formula of [XM_12_O_40_]^*n*−^, where X denotes a heteroatom (e.g., P, Si, B), M is a transition metal addenda atom (e.g., V, Mo, W), and *n* is the charge on the cluster surface.^2^ A key feature of Keggin-type POMs is their ability to incorporate various heteroatoms into their structure that can influence their redox characteristics, specifically the reduction potential, number of redox events accessible in a solvent window, and reversibility of the redox processes. These features can in turn introduce new functionalities and modular reactivity for the design of novel catalysts,^3,4^ hybrid architectures,^5,6^ redox mediators,^7,8^ and nanostructured materials.^9,10^

For the effective use of POMs in electrochemical applications, a precise control of their redox properties is critical. As such, a detailed understanding of the thermodynamics and kinetics of charge transfer in POM-based systems is imperative to fine-tune the reactivity and selectivity of these processes. In some instances, the free energy barrier to redox reactions can be directly correlated to the rates of electron transfer to and from the electrode surface.^11^ However, a large number of reactions involving POMs are solution-based, which involve supporting ions and solvent molecules. These components can alter the solvation structure around the POM through noncovalent interactions, which can result in greater energetic penalties due to solvent reorganization than are often anticipated during electron transfer.^12−16^ Thus, a correlation between the thermodynamics and kinetics of charge-transfer processes is not always apparent.^17^

A thorough knowledge of entropic contributions to the thermodynamics of charge transfer as a function of the solvation medium can aid in an accurate prediction of structure–property relationships of POMs and help identify suitable solution conditions for targeted applications. While many studies pertaining to the elucidation of the structural perturbations arising during electron-transfer processes in transition metal complexes are limited to aqueous solutions, quantification of redox entropies of anionic redox couples in nonaqueous solvents is lacking. This makes it difficult to realize the full potential of POMs for diverse applications in an array of solvents.

Herein, we report the influence of cluster charge and the properties and arrangement of heteroatom dopants within the Keggin core in polyoxotungstates on rate constants of electron transfer and changes in entropies during redox processes. We demonstrate that redox entropies of heteroatom-doped (P, V) polyoxotungstates are primarily dictated by the change in overall charge of the cluster anions in the redox reaction. In contrast, the electron-transfer rate constants are governed by the identity of the heteroatom dopant in the POM architecture. The fundamental understanding of the thermodynamics and kinetics of electron transfer in heteroatomic polyoxotungstates gained from this study will provide insights that may facilitate the targeted application of suitable POMs in the fields of catalysis, energy storage, and molecular electronics.

## Experimental Section

### General Considerations

Unless otherwise specified, the manipulations described below were carried out in the absence of water and oxygen in an MBraun UNIlab glovebox under a dinitrogen atmosphere at ambient temperature. Glassware was oven-dried for a minimum of 4 h and cooled in an evacuated antechamber prior to use. Acetonitrile was dried and deoxygenated on a Glass Contour System (Pure Process Technology, LLC) and stored over activated 3 Å molecular sieves purchased from Fisher Scientific. Tetrabutylammonium hexafluorophosphate, (^*n*^Bu_4_N)(PF_6_), was purchased from Sigma-Aldrich and recrystallized three times using hot methanol and stored under dynamic vacuum for a minimum of 2 days prior to use. (^*n*^Bu_4_N)_3_[PW_12_O_40_] (**PW**_**12**_), (^*n*^Bu_4_N)_3_[VW_12_O_40_] (**V**_**in**_**W**_**12**_), (^*n*^Bu_4_N)_4_[VW_12_O_40_], (^*n*^Bu_4_N)_4_[P(VW_11_)O_40_] (**PV**_**out**_**W**_**11**_), (^*n*^Bu_4_N)_5_[P(VW_11_)O_40_], and (^*n*^Bu_4_N)_4_[V(VW_11_)O_40_] (**V**_**in**_**V**_**out**_**W**_**11**_) were synthesized according to literature procedures.^18−20^ The radii of gyration for **PW**_**12**_, **V**_**in**_**W**_**12**_, **PV**_**out**_**W**_**11**_, and **V**_**in**_**V**_**out**_**W**_**11**_ were calculated using Multiwfn 3.8(dev) by adopting the crystal structures of the Keggin clusters.^21,22^

### Cyclic Voltammetry Measurements

Cyclic voltammetry (CV) measurements were performed using a three-electrode setup inside a dinitrogen-filled glovebox (MBraun UNIlab) using a BioLogic SP-150 potentiostat/galvanostat and the EC-Lab software suite. The concentration of clusters **PW**_**12**_ and **V**_**in**_**V**_**out**_**W**_**11**_ was kept at 1 mM and that of **V**_**in**_**W**_**12**_ and **PV**_**out**_**W**_**11**_ was kept at 2.5 mM due to solubility constraints. The supporting electrolyte (^*n*^Bu_4_N)(PF_6_) was used in 100 mM concentration for all measurements. CVs were recorded using a 3 mm diameter glassy carbon working electrode (CH Instruments, Inc.), a Pt wire auxiliary electrode (CH Instruments, Inc.), and a Ag/AgNO_3_ nonaqueous reference electrode (BASi) filled with an acetonitrile solution containing 0.01 M AgNO_3_ and 0.1 M (^*n*^Bu_4_N)(PF_6_). CVs were *iR* compensated at 95% with impedance taken at 100 kHz using the ZIR tool within the EC-Lab software. The remaining 5% uncompensated resistance was accounted for by manual correction.

### Electrokinetics Measurements

The diffusion coefficient (*D*_0_) associated with each redox process was determined at ambient temperature (∼25 °C) from variable scan rate (10 to 10000 mV s^–1^) CV data using Randles–Ševčík analysis.^23,24^ Specifically, the diffusion coefficients for the cathodic and anodic redox waves were estimated using the slopes of linear fits to the plots of peak current *i*_p_ (*i*_p,c_ and *i*_p,a_ for the cathodic and anodic waves, respectively) vs the square root of scan rate (*v*^1/2^). For a reversible redox couple, the peak current is given by eq 1:
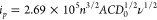
1

In eq 1, *n* is the number of electrons transferred in the reaction, *A* is the geometric electrode surface area (0.0707 cm^2^ for the glassy carbon working electrode), *C* is the bulk concentration of the redox-active species, *D*_0_ is the diffusion coefficient of the redox-active species, and ν is the scan rate. For an irreversible redox couple, the peak current is given by eq 2:
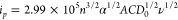
2where α is the charge-transfer coefficient. For this study, α is assumed to be 0.5 owing to the electrochemical symmetry of the investigated redox couples. For redox couples that show quasi-reversible kinetics, relationships for both reversible and irreversible redox reaction are often employed to determine the diffusion coefficients. Therefore, an average value of diffusion coefficients from eqs 1 and 2 was approximated for the quasi-reversible redox couples in this study and employed for estimation of the electron-transfer rate constants (vide infra).^25−27^

The electron-transfer kinetics were estimated directly from the variable scan rate CV data by using the Nicholson method for quasi-reversible redox reactions.^28^ The potential difference (Δ*E*_p_) of oxidation and reduction peaks was obtained at different scan rates. The transfer parameter (ψ) was extracted from the working curve constructed by Nicholson using Δ*E*_p_ values. The standard heterogeneous electron-transfer rate constant (*k*_0_) for a given reduction process was determined using eq 3:
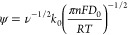
3where *n* is the number of electrons transferred in the reaction, *F* is Faraday’s constant, *v* is the scan rate, *D*_0_ is the average diffusion coefficient (determined by employing eqs 1 and 2) obtained from the slope of *i*_p,c_ vs *v*^1/2^ plots, *R* is the ideal gas constant, and *T* is the temperature.

### Temperature Coefficient Measurements of Polyoxotungstates

Measurements were conducted under a dinitrogen atmosphere using a BioLogic SP-150 potentiostat in an isothermal electrochemical setup where the temperature was controlled using a hot plate. The actual temperature near the working electrode was monitored using a thermocouple (stainless steel), which was calibrated using an external temperature controller (Omega Engineering, CS8DPT). Glassy carbon (3 mm diameter, CH Instruments, Inc.) and Pt wire (CH Instruments, Inc.) were used as the working and auxiliary electrodes, respectively. Potentials are referenced to a Ag/AgNO_3_ nonaqueous reference electrode (BASi) filled with an acetonitrile solution containing 0.01 M AgNO_3_ and 0.1 M (^*n*^Bu_4_N)(PF_6_). For **V**_**in**_**W**_**12**_, the temperature coefficients of the redox couples were evaluated using both isothermal open circuit potential (OCP) and CV measurements, whereas only CV measurements were used for the other studied polyoxotungstate clusters.

In the OCP method, 2 mL each of the oxidized (1 mM (^*n*^Bu_4_N)_3_[VW_12_O_40_] in acetonitrile containing 0.1 M (^*n*^Bu_4_N)(PF_6_)) and reduced (1 mM (^*n*^Bu_4_N)_4_[VW_12_O_40_] in acetonitrile containing 0.1 M (^*n*^Bu_4_N)(PF_6_)) cluster solutions were mixed together. The temperature of the cell was increased in 4–6 °C intervals in the temperature range 19–55 °C and recorded near the working electrode using a thermocouple. The temperature readings of the thermocouple were calibrated using an external temperature controller. At each temperature, the OCP was recorded for 120 s. Solutions were stirred between measurements at different temperatures. The average OCP values were plotted against temperature, and the slope of the linear fit to the data provided the temperature coefficient of the [VW_12_O_40_]^3–^/[VW_12_O_40_]^4–^ redox couple. The reported error corresponds to the standard deviation of three independent measurements.

In the CV method, a 1 mM solution of the corresponding polyoxotungstate (**PW**_**12**_, **V**_**in**_**W**_**12**_, **PV**_**out**_**W**_**11**_, and **V**_**in**_**V**_**out**_**W**_**11**_) in acetonitrile containing 0.1 M (^*n*^Bu_4_N)(PF_6_) was used for estimating the temperature coefficients. The cell and electrode setup conditions were mirrored from the aforementioned isothermal variable temperature OCP (VT-OCP) measurements. The scan rate of each measurement was set to 100 mV s^–1^ and positive scan direction was used. The temperature of the cell was increased in 4–6 °C intervals in the temperature range 19–55 °C and recorded near the working electrode using a thermocouple. The temperature readings of the thermocouple were calibrated using an external temperature controller. At each temperature value, the cell was allowed to equilibrate for ∼5 min before recording the CVs. Solutions were stirred between measurements at different temperatures. The *E*_1/2_ values for each redox couple of every polyoxotungstate cluster were extracted from the VT-CV data and plotted against temperature. The slopes of the linear fits to the data correspond to the temperature coefficients of the redox couples. Note that all temperature coefficients are reported in units of mV K^–1^, as is the convention in the field. These values were used to estimate the redox reaction entropies (Δ*S*_redox_) of the studied polyoxotungstates (see Supporting Information for sample calculation).^29^ The reported errors correspond to the standard deviation of three independent measurements for each cluster.

### Temperature Coefficient Measurements of Reference Electrode

The temperature coefficient of the Ag/AgNO_3_ nonaqueous reference electrode potential was estimated using nonisothermal OCP measurements in a two-electrode setup following a modified literature procedure, where one Ag/AgNO_3_ reference electrode serves as a working electrode and a second Ag/AgNO_3_ reference electrode serves as a reference/auxiliary electrode.^12^ A three-compartment custom-made glass cell (Adams & Chittenden Scientific) with fine frits separating the two side compartments from the middle compartment was washed with concentrated nitric acid (ACS Plus, 15.8 N, Fisher Scientific) and deionized water between experiments, and oven-dried for at least 1 h prior to use. The cell was arranged such that each side compartment was immersed in a separate oil bath. For each measurement, ∼10 mL and ∼3 mL of acetonitrile containing 0.1 M (^*n*^Bu_4_N)(PF_6_) were added to the side and middle compartments, respectively. The solutions were stirred and purged with argon gas for 10 min. Subsequently, each side compartment was charged with a Ag/AgNO_3_ reference electrode (CH Instruments, Inc.) filled with an acetonitrile solution containing 0.01 M AgNO_3_ and 0.1 M (^*n*^Bu_4_N)(PF_6_), and a thermocouple (stainless steel) arranged at the same height. The electrodes were allowed to equilibrate for 5 min while purging with argon gas prior to data collection. VT-OCP measurements were conducted using CH Instruments 760E electrochemical workstation by maintaining the side compartment housing the Ag/AgNO_3_ electrode serving as reference/auxiliary electrode at ambient temperature and the temperature of the side compartment housing the Ag/AgNO_3_ electrode serving as working electrode was varied from ∼25 °C to ∼45 °C in ∼3 °C intervals. The temperature readings of the thermocouples were calibrated using an external temperature controller (Omega Engineering, CS8DPT). At each temperature, the OCP was recorded for 60 s with stirring and argon gas flowing in the headspace. The average OCP values were plotted against the temperature difference between the two Ag/AgNO_3_ electrodes, and the slope of the linear fit to the data provided the temperature coefficient of the Ag/AgNO_3_ reference electrode potential. The reported value of +0.43(6) mV K^–1^ is an average obtained from four independent experiments. This value is in good agreement with values previously reported for the Ag/AgNO_3_ reference electrode potential in acetonitrile solutions containing other electrolytes.^30^ To estimate the error associated with the measured temperature coefficient value, we repeated the VT-OCP measurements using different sets of Ag/AgNO_3_ electrodes as working and reference/auxiliary electrodes and swapped their location (i.e., heated vs nonheated compartments). The corresponding temperature coefficient values ranged from +0.38 to +0.45 mV K^–1^, thus an average value of +0.43 mV K^–1^ with an error of 0.06 mV K^–1^ is reported. We note that no correction for the thermal liquid junction potential was made as such contribution should be minimal in a three-compartment double-fritted electrochemical cell using the same electrolyte solution inside and outside of the reference electrode frit.

## Results and Discussion

### Effect of Heteroatom Substitution on Kinetics of Charge Transfer

**Figure 1:**
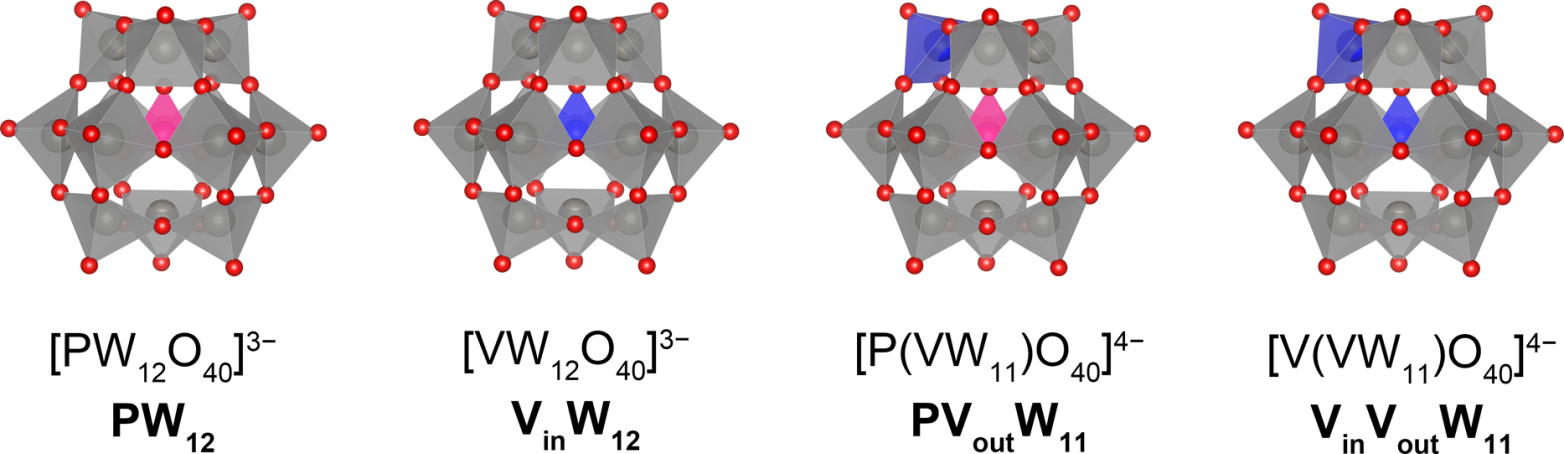
Structures of polyoxotungstates with different central and surface heteroatoms studied in the present work: **PW**_**12**_, **V**_**in**_**W**_**12**_, **PV**_**out**_**W**_**11**_, and **V**_**in**_**V**_**out**_**W**_**11**_. Gray, blue, and pink polyhedra denote W, V, and P atoms, respectively, and the red spheres represent O atoms.

An attractive characteristic of Keggin-type POMs for energy-related applications is their ability to undergo multiple electron-transfer reactions without significant restructuring of the cluster framework.^1^ The change in coordination environment of a metal center in POMs, through ligand modification and/or neighboring metal substitution, can significantly influence its charge-transfer behavior. In particular, heteroatom doping can be leveraged to yield isomorphous, isostructural, and isocharge congeners of a parent cluster with contrasting electronic properties.^31^ As such, we became interested in quantifying the impact of doping on the rate constants of electron transfer in four Keggin-type polyoxotungstates: [PW_12_O_40_]^3–^ (**PW**_**12**_), [VW_12_O_40_]^3–^ (**V**_**in**_**W**_**12**_), [P(VW_11_)O_40_]^4–^ (**PV**_**out**_**W**_**11**_), and [V(VW_11_)O_40_]^4–^ (**V**_**in**_**V**_**out**_**W**_**11**_) (Figure 1). These clusters were selected owing to their tunable structural and electronic properties, allowing us to study the effect of the identity and the location of the dopant on the kinetics of charge transfer in multimetallic systems.

We began our investigations by comparing the centrally substituted polyoxotungstates, **PW**_**12**_ and **V**_**in**_**W**_**12**_. Our motivation behind this comparison stems from our hypothesis that the nature of the dopant (i.e., redox-innocent vs redox-active) will influence the kinetics of charge transfer for W-based events. The structure of **PW**_**12**_ is reported in the literature.^32−34^ It consists of a pentavalent, central phosphorus atom with 12 addenda tungstate ions bridged through shared oxide ligands. The spatial location of the atoms within the Keggin core dictates their coordination environment. In **PW**_**12**_, the P^V^ atom is present in a tetrahedral coordination environment at the center of the cluster, whereas the framework W^VI^ atoms are in an octahedral ligand field (Figure 1). Isovalent doping of the phosphorus atom with vanadium results in the formation of [VW_12_O_40_]^3–^ (**V**_**in**_**W**_**12**_), where both V and W atoms are in their most oxidized state (+5 and +6, respectively) (Figure 1). Replacement of P^V^ with V^V^ inside the Keggin core results in an increase in the average bond length between the central atom and oxygen, while the bonds between tungsten and oxygen atoms attached to the central atom remain constant (Table 1).^35^ This behavior can be ascribed to the difference in the effective ionic radius of tetrahedrally coordinated P^V^ ion (0.31 Å) as compared to V^V^ (0.50 Å).^36^

**Table 1:** Selected Mean Interatomic Bond Distances (Å) in Polyoxotungstates with the General Formula XMW_11_ (X = Central Heteroatom = V or P; M = Addenda Atom = V or W)^a^

**POM**	**X–O**_**c**_	**M–O**_**c**_	**M–O**_**b**_	**M = O**_**t**_	**Reference**
**PW**_**12**_	1.52(2)	2.46(2)	1.89(1)	1.68(1)	(18)
**V**_**in**_**W**_**12**_	1.57(1)	2.46(2)	1.89(2)	1.68(1)	(35)
**PV**_**out**_**W**_**11**_	1.52(2)	2.46(1)	1.89(1)	1.66(1)	(41)
**V**_**in**_**V**_**out**_**W**_**11**_	1.65(2)	2.39(2)	1.87(2)	1.63(2)	(42)

a O_c_ represents an O atom bonded to a central heteroatom, O_b_ denotes a bridging O atom, and O_t_ denotes a terminal O atom.

**Figure 2:**
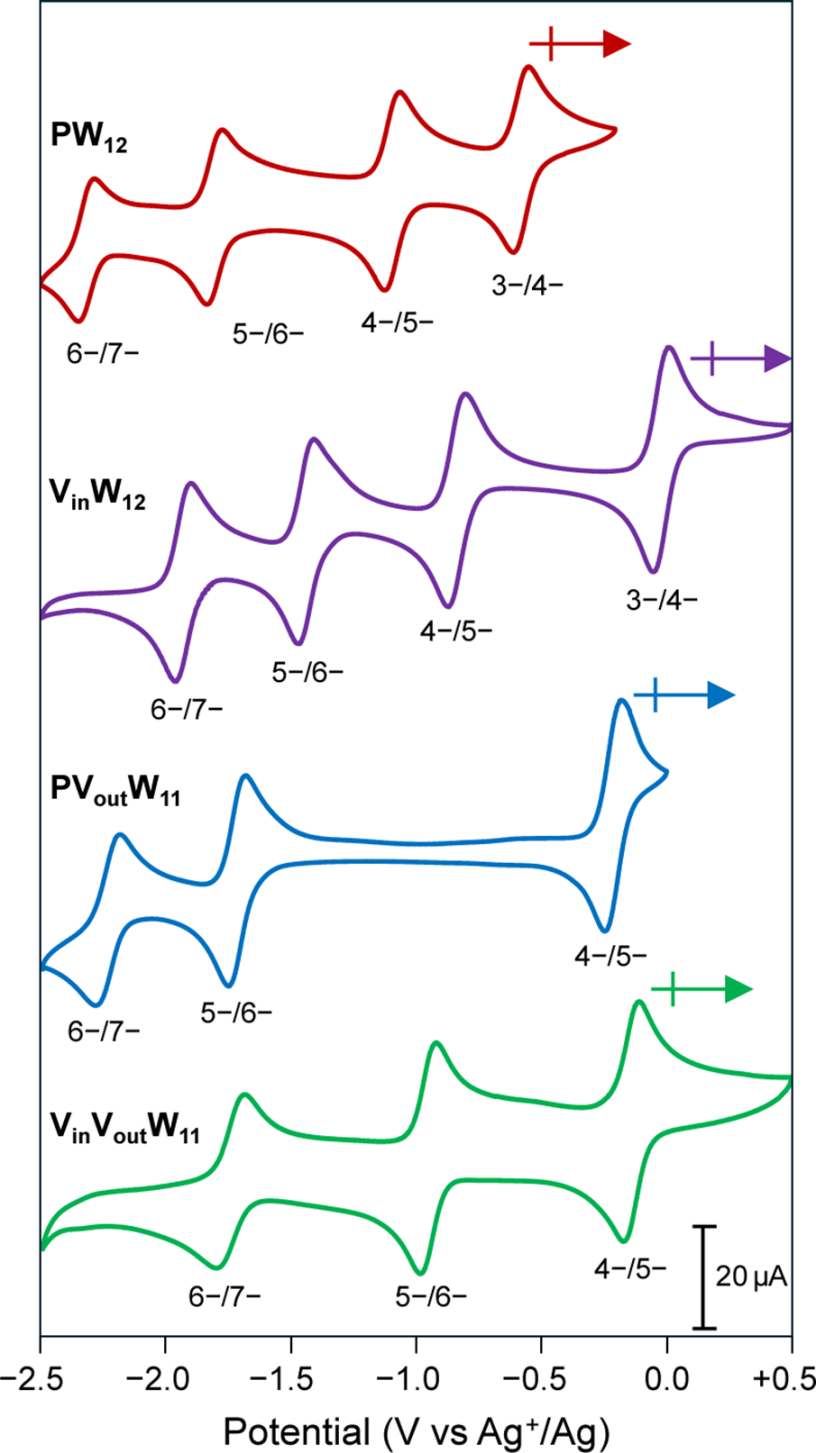
Cyclic voltammograms of the studied polyoxotungstates: **PW**_12_ (red), **V**_in_**W**_12_ (purple), **PV**_out_**W**_11_ (blue), and **V**_in_**V**_out_**W**_11_ (green). All voltammograms were collected at ambient temperature using glassy carbon working electrode, Ag/AgNO_3_ reference electrode, and Pt auxiliary electrode in acetonitrile with 0.1 M (^*n*^Bu_4_N)(PF_6_) as the supporting electrolyte at a scan rate of 100 mV s^–1^. The arrows indicate the open circuit potential and scan direction. The negative numbers below the voltammograms denote the charge states corresponding to each redox wave.

As substitution of the central atom results in significant structural changes within the Keggin core, we sought to probe how they impact the electronic properties of the polyoxotungstate cluster. To assess the electronic properties of **PW**_**12**_ and **V**_**in**_**W**_**12**_, cyclic voltammograms (CVs) were collected in acetonitrile with 0.1 M (^*n*^Bu_4_N)(PF_6_) as the supporting electrolyte in the potential window −2.5 V to +0.5 V vs Ag^+^/Ag—a window in which the phosphorus atom in **PW**_**12**_ is redox inactive. The voltammogram of **PW**_**12**_ reveals four quasi-reversible, one-electron redox events located at *E*_1/2_ values of −0.58 V, –1.10 V, –1.81 V, and −2.31 V vs Ag^+^/Ag (Figure 2, **red**). Each of these redox events corresponds to the formal reduction of a single W^VI^ atom. However, it has been reported that the unpaired electrons generated upon reduction of the tungsten sites are delocalized over the 12 tungsten atoms.^37,38^ The CV of **V**_**in**_**W**_**12**_ similarly displays four redox processes located at *E*_1/2_ values of −0.02 V, –0.83 V, –1.44 V, and −1.93 V vs Ag^+^/Ag (Figure 2, purple). However, characterization of the one-electron reduced product of **V**_**in**_**W**_**12**_ shows that the location of cluster reduction is different for the first and subsequent reduction events.^19^ The first reducing equivalent (*E*_1/2_ = −0.02 V vs Ag^+^/Ag) goes to the vanadium atom embedded within the Keggin core (V^V^/V^IV^),^19^ whereas the subsequent reductions are assigned to the formal reduction of tungsten atoms (W^VI^/W^V^).^19^ The different redox properties of **PW**_**12**_ and **V**_**in**_**W**_**12**_ prompted us to investigate the influence of the central atom on the kinetics of heterogeneous electron transfer of the Keggin assembly, in particular the rate of W-based reductions.

To determine the electrokinetic behavior of the **PW**_**12**_ and **V**_**in**_**W**_**12**_ clusters, we first evaluated their diffusion coefficients (*D*_0_) via Randles–Ševčík analysis.^23,24^ Each of the redox couples was isolated and CVs were recorded at scan rates ranging from 10 to 10000 mV s^–1^. Plots of the cathodic (*i*_p,c_) or anodic peak current (*i*_p,a_) vs the square root of scan rate (ν^1/2^) exhibit a linear dependence for all redox couples, consistent with diffusion-limited electrochemical processes (Figures S1–S6). The obtained diffusion coefficients for all redox couples are summarized in Tables S1 and S2. Using the cathodic diffusion coefficients, we then estimated the rate constants (*k*_0_) of cluster reductions using the Nicholson method (Figures S7–S12).^28^ The one-electron reduction of **PW**_**12**_ (3–/4−) exhibits a *k*_0_ value of 0.19(2) cm s^–1^, whereas the *k*_0_ values for the 4–/5– and 5–/6– reductions are estimated to be 0.18(4) cm s^–1^ and 0.046(2) cm s^–1^, respectively. As discussed above, these events are assigned to W-based reductions. The slower reduction rate for the 5–/6– redox couple may be attributed to the additional kinetic barrier that can arise for the isomerization of α-**PW**_**12**_ to β-**PW**_**12**_ after the second reduction.^39,40^ Similar analysis of **V**_**in**_**W**_**12**_ yields a rate constant of 0.10(4) cm s^–1^ for the V-based reduction (**V**_**in**_**W**_**12**_ 3–/4−) and the subsequent W-based one-electron reductions afford *k*_0_ values of 0.035(3) cm s^–1^ and 0.11(2) cm s^–1^ for the 4–/5– and 5–/6– reductions, respectively. While the reason for the slower kinetics of the 4–/5– redox couple as compared to the 5–/6– couple is currently unknown, we postulate that it may either be credited to structural changes, such as α/β isomerization, as observed for **PW**_**12**_ (vide supra),^39^ and/or changes in the electronic structures across the respective oxidation states, as suggested by DFT studies on related Keggin-type heteroatom-doped polyoxotungstates.^40^ The estimated values of *k*_0_ are summarized in Table 2.

**Table 2:** Heterogeneous Electron-Transfer Rate Constants^a^for One-Electron Reductions of **PW**_12_, **V**_in_**W**_12_, **PV**_out_**W**_11_, and **V**_in_**V**_out_**W**_11_, as Calculated Using the Nicholson Method^28^

**POM**	**Charge state**	**Redox couple**	**Rate constant**, *k*_**0**_ (cm s^–1^)
**PW**_**12**_	3–/4–	W^VI^/W^V^	0.19(2)
	4–/5–	W^VI^/W^V^	0.18(4)
	5–/6–	W^VI^/W^V^	0.046(2)
**V**_**in**_**W**_**12**_	3–/4–	V_in_^V^/V_in_^IV^	0.10(4)
	4–/5–	W^VI^/W^V^	0.035(3)
	5–/6–	W^VI^/W^V^	0.11(2)
**PV**_**out**_**W**_**11**_	4–/5–	V_out_^V^/V_out_^IV^	0.07(1)
	5–/6–	W^VI^/W^V^	0.08(3)
**V**_**in**_**V**_**out**_**W**_**11**_	4–/5–	V_out_^V^/V_out_^IV^	0.12(1)
	5–/6–	V_in_^V^/V_in_^IV^	0.14(3)

a Note that the average *D*_0_ values obtained from the reversible and irreversible Randles–Ševčík eqs (eqs 1 and 2) for the cathodic peak currents (Table S1) were employed to calculate all *k*_0_ values. The reported errors correspond to the standard error associated with each data set (see Supporting Information for relevant plots of ψ vs *v*^–1/2^) in the 95% confidence interval of the regression analysis.

The observed values of *k*_0_ in **PW**_**12**_ and **V**_**in**_**W**_**12**_ suggest that there is a decrease in the rate of the first W-based reduction upon incorporation of a redox-active central heteroatom in the Keggin core. It should be noted that while the change is small when absolute values are compared, the effect of the identity of the central heteroatom on the kinetics of charge transfer in Keggin-type polyoxotungstates cannot be ignored. This prompted us to experimentally evaluate the electron-transfer rate constants of **PV**_**out**_**W**_**11**_. Our motivation behind selecting this cluster is rooted in the rationale that comparing the electrokinetics of **PW**_**12**_, **PV**_**out**_**W**_**11**_, and **V**_**in**_**W**_**12**_ would not only yield information about the influence of the *identity* of the heteroatom on the charge-transfer propensity of polyoxotungstates, but also the spatial *location* of the dopant.

The structure of **PV**_**out**_**W**_**11**_ is shown in Figure 1. Similar to **V**_**in**_**W**_**12**_, the V atom in **PV**_**out**_**W**_**11**_ is in a +5 oxidation state and all W atoms are in the +6 oxidation state. The only significant structural difference between **PV**_**out**_**W**_**11**_ and **V**_**in**_**W**_**12**_ lies in the X–O_c_ bond distances; P–O bonds are shorter than V–O bonds (Table 1).^41^ Thus, **PV**_**out**_**W**_**11**_ more closely resembles the molecular structure of **PW**_**12**_. Characterization of **PV**_**out**_**W**_**11**_ via CV reveals three quasi-reversible, one-electron redox events between −2.5 V and +0.5 V vs Ag^+^/Ag with *E*_1/2_ values of −0.21 V, –1.72 V, and −2.22 V vs Ag^+^/Ag (Figure 2, **blue**). This electrochemical behavior is significantly different from that observed for **V**_**in**_**W**_**12**_, which exhibits four redox events in the same potential window. The reduction event at −0.21 V vs Ag^+^/Ag is assigned to the V^V^/V^IV^ reduction,^20^ whereas the events at more negative potentials can be ascribed to the sequential reduction of two tungsten sites (W^VI^/W^V^).^20^ The diffusion coefficients of the cluster for the 4–/5– and 5–/6– redox couples resemble those obtained for **V**_**in**_**W**_**12**_ (Figures S13 and S14). Nicholson analysis of the reduction events of **PV**_**out**_**W**_**11**_ reveals that the one-electron V-based reduction of [P(VW_11_)O_40_]^4–^ (4–/5−) has a *k*_0_ value of 0.07(1) cm s^–1^, while the first W-based reduction event (5–/6−) affords a *k*_0_ value of 0.08(3) cm s^–1^ (Figures S15 and S16). These results indicate that the rate of the first W-based reduction in **PV**_**out**_**W**_**11**_ is statistically similar to the rates of W-based reductions in **V**_**in**_**W**_**12**_, and both are slower than in **PW**_**12**_. These observations suggest that V-doping leads to a similar drop in reduction rates regardless of the location of the heteroatom in Keggin-type polyoxotungstates. This behavior is contrary to our expectations as the two V atoms are in significantly different coordination environments—the addenda **V**_**out**_ is in octahedral geometry whereas the central **V**_**in**_ is tetrahedrally coordinated.

Our results on the electrode kinetics of **V**_**in**_**W**_**12**_ and **PV**_**out**_**W**_**11**_ reveal that the rates of V-based reductions are independent of the spatial location of the dopant. This observation prompted the question whether the kinetic behavior of the V atoms is retained upon introducing multiple redox-active dopants in the same assembly. To probe this question, we evaluated the electrochemical properties of **V**_**in**_**V**_**out**_**W**_**11**_. This cluster features two pentavalent vanadium atoms and all tungsten atoms are fully oxidized, i.e., W^VI^ (Figure 1). While the average V(central)–O_c_ bond length is greater in **V**_**in**_**V**_**out**_**W**_**11**_ as compared to the other polyoxotungstates studied herein (**PW**_**12**_, **V**_**in**_**W**_**12**_, and **PV**_**out**_**W**_**11**_), the average bond distances between the addenda atoms and surrounding oxygen atoms are comparatively short (Table 1).^42^

The CV of **V**_**in**_**V**_**out**_**W**_**11**_ resembles that of **PV**_**out**_**W**_**11**_ as both clusters display three quasi-reversible, one-electron redox events in the studied potential range (Figure 2, green and blue). The first redox event at *E*_1/2_ = −0.14 V vs Ag^+^/Ag is ascribed to a V^V^/V^IV^ redox couple of the *addenda* vanadium atom (labeled **V**_**out**_) in the Keggin cluster.^19^ The second redox event for **V**_**in**_**V**_**out**_**W**_**11**_ (*E*_1/2_ = −0.95 V vs Ag^+^/Ag) is assigned to a V^V^/V^IV^ redox couple of the *central* vanadium atom (labeled **V**_**in**_).^19^ The reason for the lower potential for **V**_**out**_ reduction in comparison to that observed for **V**_**in**_ is that when an addenda W atom is substituted with V, the doubly degenerate LUMO in XW_12_ (X = heteroatom) splits in two orbitals, such that the lowest unoccupied orbital has more **V**_**out**_ character.^31^ The third redox feature with *E*_1/2_ = −1.77 V vs Ag^+^/Ag is assigned to the first W-based redox event (W^VI^/W^V^) of the Keggin cluster. Despite possessing the same charge state, there is a considerable shift in the *E*_1/2_ values toward less negative potentials for **V**_**in**_**V**_**out**_**W**_**11**_ as compared to **PV**_**out**_**W**_**11**_. For the 4–/5– redox couple, this behavior can be ascribed to the differing site of reduction in **V**_**in**_**V**_**out**_**W**_**11**_ and **PV**_**out**_**W**_**11**_; W-based (5d metal) reductions generally occur at more negative potentials than those observed for V (3d metal).^31,43^ With this understanding of the electrochemical behavior of the doubly doped polyoxotungstate, we turned our attention to probing its electrokinetic properties.

The rate constants of electron transfer for sequential one-electron reductions of **V**_**in**_**V**_**out**_**W**_**11**_ associated with the redox couples 4–/5– and 5–/6– are 0.12(1) cm s^–1^ and 0.14(3) cm s^–1^, respectively (Table 2, Figures S17–S20). The similar values for V-based reductions in **V**_**in**_**W**_**12**_, **PV**_**out**_**W**_**11**_, and **V**_**in**_**V**_**out**_**W**_**11**_ suggest that neither the location nor the number of dopant atoms significantly impact the kinetics of charge transfer in this series of Keggin assemblies.

Overall, our results on the electrokinetics of **PW**_**12**_, **V**_**in**_**W**_**12**_, **PV**_**out**_**W**_**11**_, and **V**_**in**_**V**_**out**_**W**_**11**_ indicate that the *presence* of a heteroatom, as opposed to spatial location or number of heteroatoms, influences the rate constants of charge transfer in polyoxotungstates. Indeed, V-doping within the Keggin framework induces slower electron transfer for W-based reduction events. It should be noted that the results presented in this study are different than those described in a recent report.^44^ Through simulation of Fourier Transformed large amplitude alternating current voltammetry, the authors report that the site and reduction levels of V-substituted polyoxometalates drastically affect the electrokinetics. In their study, the predicted rates of reduction of the central **V**_**in**_ and addenda **V**_**out**_ in **V**_**in**_**V**_**out**_**W**_**11**_ are demonstrated to be distinct, albeit no error values are provided. However, according to the authors, while the trend approximately holds true when comparing the **V**_**out**_ kinetics in **V**_**in**_**V**_**out**_**W**_**11**_ with those observed for [SV_out_W_11_O_40_]^3–^, this argument fails for the central **V**_**in**_ atom in **V**_**in**_**W**_**12**_ and hints toward the possible role of overall charge on the cluster surface in influencing the kinetic behavior. As such, the impetus of the electron-transfer ability of V-doped POMs remains elusive. To better understand this conundrum, we focused our attention toward identifying the correlation between the thermodynamic driving force and rates of electron transfer using electrochemical entropy measurements.

### Entropy of Redox Reactions

In addition to structural and electronic differences, the redox kinetics can also be influenced by the solvation structure around an ion.^45−47^ Often times, the role of ternary interactions between the redox-active species, solvent molecules, and supporting electrolyte ions are ignored while sketching a holistic picture of the electron-transfer kinetics.^48^ Thus, to better understand the role of redox-innocent components in perturbing the solvation structure around the polyoxotungstate clusters, we sought to evaluate the entropic changes associated with the charge-transfer reactions. Several literature reports have alluded to the efficacy of electrochemical measurements in quantifying the entropic barriers to electron transfer.^12,14,16,29,49^ Therefore, we reasoned that this thermodynamic property would yield information about the energetic losses associated with structural reorganization during electron transfer to and from the electrode surface.

The change in entropy associated with a charge-transfer reaction (Δ*S*_redox_) is the difference in entropies between the reduced (*S*_red_) and oxidized (*S*_ox_) forms of a redox-active species. They are correlated to the formal potential or half-wave potential (*E*_1/2_) as described in eq 4:
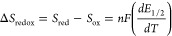
4Here, *n* is the number of electrons transferred in the redox reaction, *F* is Faraday’s constant, and *T* is the solution temperature. In our study, the formal potential (*E*^0^′) can be estimated as the half-wave potential (*E*_1/2_) owing to the similar diffusion coefficients of the oxidized and reduced polyoxotungstate species (Tables S1 and S2). As Δ*S*_redox_ values provide information about the reorganization energies that are involved in redox processes, we evaluated entropic changes in the series of heteroatom-substituted polyoxotungstates using two different electrochemical approaches: (1) the open circuit potential (OCP) method,^12^ and (2) the CV method.^14^

**Figure 3:**
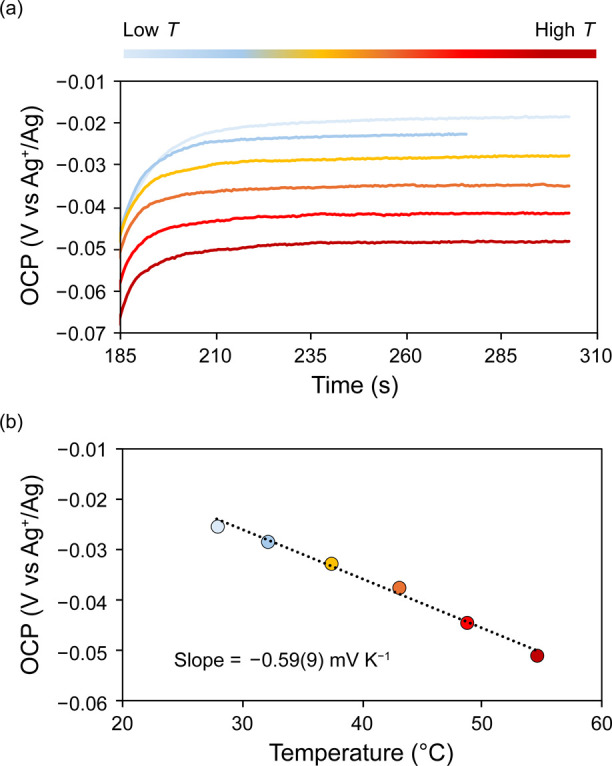
(a) A representative example of data obtained from a VT-OCP measurement of an equimolar solution (1 mM each) of 3– and 4– redox states of **V**_**in**_**W**_**12**_ in acetonitrile with 0.1 M (^*n*^Bu_4_N)(PF_6_) as the supporting electrolyte using isothermal electrochemical setup from a low temperature of 27.8 °C (blue) to a high temperature of 54.4 °C (red). (b) Observed temperature dependence of the corresponding OCP values at steady state. The reported slope is an average of three independent measurements and the error in the slope corresponds to the standard deviation obtained from these measurements. The VT-OCP measurements were collected using glassy carbon working electrode, Ag/AgNO_3_ reference electrode, and Pt auxiliary electrode in an isothermal single-compartment cell.

For the OCP method, we chemically isolated the **V**_**in**_**W**_**12**_ cluster in the 3– (most oxidized state) and 4– (one-electron reduced state) charge states. Equimolar solutions (1 mM each) of the 3– and 4– charge states of **V**_**in**_**W**_**12**_ in acetonitrile containing 0.1 M (^*n*^Bu_4_N)(PF_6_) as a supporting electrolyte were used to evaluate the OCP at different temperatures. The measured OCP values correspond to the formal potentials of the 3–/4– redox couple, which can be estimated as *E*_1/2_ owing to the similar diffusion coefficients of the oxidized and reduced polyoxotungstate species (Tables S1 and S2). The temperature-induced shift in *E*_1/2_, known as the temperature coefficient, is correlated to the redox entropy of the reaction as shown in eq 4.^12^Figure 3 shows a representative example of the experimentally obtained OCP traces of the 3–/4– redox couple at temperatures ranging from 19 to 55 °C. A negative shift in OCP is observed with increasing temperature, consistent with an anionic redox couple.^50,51^ To evaluate the associated redox entropy, the temperature coefficient of the Ag/AgNO_3_ reference electrode potential (+0.43(6) mV K^–1^) was quantified in a nonisothermal experimental setup and used for Δ*S*_redox_ calculations (see Supporting Information for sample calculation, Figure S21). The temperature coefficient of the 3–/4– couple of **V**_**in**_**W**_**12**_ is evaluated to be −0.59(9) mV K^–1^ using the OCP method, corresponding to Δ*S*_redox_ = −57(6) J K^–1^ mol^–1^. While the literature lacks in quantitative estimation of Δ*S*_redox_ values of anionic redox couples in acetonitrile, comparing these values to those reported for the Wells–Dawson ion, [P_2_W_18_O_62_]^6–^, in *N*,*N*-dimethylformamide reveals that the Keggin POM, **V**_**in**_**W**_**12**_, has a lower absolute temperature coefficient than the 7–/8– and 8–/9– redox couples of the Wells–Dawson cluster,^49^ which may arise from the different charges of these POMs. A similar trend is observed when comparing our obtained values to those reported for [Fe(CN)_6_]^3–/4–^ in aqueous solutions.^52^ This discrepancy can be attributed to a combined effect of differences in size and solvation around the POM and iron complex, despite possessing similar charges.

**Figure 4:**
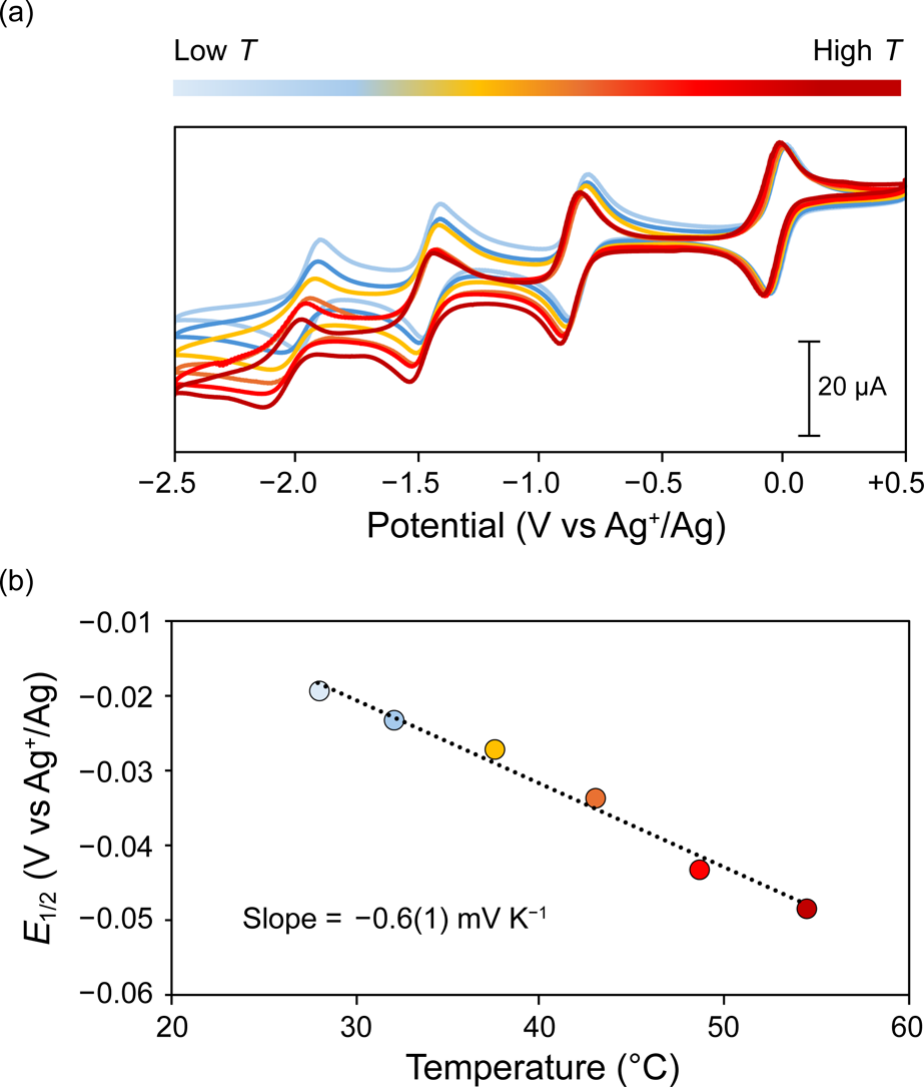
(a) A representative example of data obtained from a VT-CV measurement of a 1 mM solution of **V**_**in**_**W**_**12**_ in acetonitrile with 0.1 M (^*n*^Bu_4_N)(PF_6_) as the supporting electrolyte using isothermal electrochemical setup from a low temperature of 27.8 °C (blue) to a high temperature of 54.4 °C (red). (b) Observed temperature dependence of the *E*_1/2_ values obtained from the corresponding CVs. The reported slope is an average of three independent measurements and the error in the slope corresponds to the standard deviation obtained from these measurements. The VT-CV measurements were collected using glassy carbon working electrode, Ag/AgNO_3_ reference electrode, and Pt auxiliary electrode in an isothermal single-compartment cell.

To determine the accuracy of the obtained values, we also estimated the redox entropy using the CV method.^14^ Similar to the OCP method, the voltammograms were collected at variable temperatures (Figure 4). The negative shift of both the anodic and cathodic waves with increasing temperature indicates a negative shift in *E*_1/2_ with increasing temperature. The *E*_1/2_ values were extracted for the 3–/4– redox couple of **V**_**in**_**W**_**12**_ and plotted against the corresponding temperatures to yield a temperature coefficient of −0.6(1) mV K^–1^ and a Δ*S*_redox_ value of −59(12) J K^–1^ mol^–1^. These values agree well with those obtained using the OCP method, thus underscoring the efficacy of both methods in estimating the redox entropies of charge-transfer reactions in polyoxotungstate compounds.

The CV method has an advantage over the OCP technique for Δ*S*_redox_ estimation as it allows for the direct measurement of the redox entropies of multiple electron-transfer reactions at once, without needing to chemically isolate the corresponding charged species. To illustrate, the VT-CVs of **V**_**in**_**W**_**12**_ shown in Figure 4 can be used to extract the half-wave potentials of the 3–/4–, 4–/5–, 5–/6–, and 6–/7– couples in a single experiment (Figure S22). We decided to use the CV method for the remainder of this study due to the ease of the experimental protocol and the accuracy of the obtained values across different electrochemical measurements.

The observed temperature coefficient for the 4–/5– redox couple of **V**_**in**_**W**_**12**_ is −0.88(8) mV K^–1^, which corresponds to a Δ*S*_redox_ value of −85(7) J K^–1^ mol^–1^ (Figure S23). These values are greater in magnitude than those obtained for the 3–/4– couple. Similarly, the temperature coefficient and redox entropy for the 5–/6– redox couple are −1.1(2) mV K^–1^ and −103(9) J K^–1^ mol^–1^, respectively, which are more negative than the corresponding values for the lower charge states of V_in_W_12_ (Figure S24). The temperature coefficients and redox entropies for the successive one-electron redox couples of **PW**_**12**_, **V**_**in**_**W**_**12**_, **PV**_**out**_**W**_**11**_, and **V**_**in**_**V**_**out**_**W**_**11**_ obtained from VT-CV measurements are summarized in Table 3 (Figures S25–S34).

**Table 3:** Temperature Coefficients and Redox Entropies for Successive One-Electron Redox Couples of **PW**_12_, **V**_in_**W**_12_, **PV**_out_**W**_11_, and **V**_in_**V**_out_**W**_11_, as Evaluated from VT-CV Measurements^a^

**POM**	**Redox couple**	**Temperature coefficient**(mV K^–1^)	**Δ***S*_**redox**_(J K^–1^ mol^–1^)
**PW**_**12**_	3–/4–	–0.5(1)	–53(11)
	4–/5–	–0.91(7)	–88(4)
	5–/6–	–1.1(2)	–104(13)
**V**_**in**_**W**_**12**_	3–/4–	–0.6(1)	–59(12)
	4–/5–	–0.88(8)	–85(7)
	5–/6–	–1.1(2)	–103(9)
**PV**_**out**_**W**_**11**_	4–/5–	–0.84(1)	–81(9)
	5–/6–	–1.1(1)	–106(12)
**V**_**in**_**V**_**out**_**W**_**11**_	4–/5–	–0.69(6)	–67(18)
	5–/6–	–1.04(7)	–100(5)

a Note that while the voltammograms exhibit multiple redox waves for each of these polyoxotungstate clusters, only those redox couples that display stable behavior across the studied temperature range are evaluated (see Figure S22 for details). The values are obtained from VT-CV analysis of 1 mM solution of cluster in acetonitrile containing 0.1 M (^*n*^Bu_4_N)(PF_6_) as the supporting electrolyte using glassy carbon working electrode, Ag/AgNO_3_ reference electrode, and Pt auxiliary electrode at a scan rate of 100 mV s^–1^ in an isothermal single-compartment cell. The reported errors correspond to the standard deviation obtained from three independent measurements.

Based on the VT-CV data, a general trend is observed for the temperature coefficient and redox entropy across the studied polyoxotungstates (Table 3). The temperature coefficient for the 3–/4– redox couple of **V**_**in**_**W**_**12**_ is −0.6(1) mV K^–1^, which is statistically identical to the −0.5(1) mV K^–1^ value obtained for the corresponding charge state of **PW**_**12**_. As a result, the two clusters exhibit similar redox entropy values of −59(12) J K^–1^ mol^–1^ and −53(11) J K^–1^ mol^–1^ for **V**_**in**_**W**_**12**_ and **PW**_**12**_, respectively. Analogously, the redox entropies for the 4–/5– redox couple of **V**_**in**_**W**_**12**_, **PW**_**12**_, and **PV**_**out**_**W**_**11**_, and the 5–/6– redox couple of **V**_**in**_**W**_**12**_, **PW**_**12**_, **PV**_**out**_**W**_**11**_, and **V**_**in**_**V**_**out**_**W**_**11**_ are within error of each other, respectively. One anomaly in our experimental results is the 4–/5– redox couple of **V**_**in**_**V**_**out**_**W**_**11**_ whose temperature coefficient deviates slightly from the general trend exhibited by the other polyoxotungstate clusters; however, the Δ*S*_redox_ value is within experimental error of the values obtained for the 4–/5– redox couple in the other three clusters. Together, these results indicate that there is a strong correlation between the experimentally obtained redox entropies, temperature coefficients, and the charge of the polyoxotungstate clusters.

The observed values of Δ*S*_redox_ are not only closely correlated to the charge of the Keggin assembly, but also the *charge density* of the clusters. Specifically, the values of Δ*S*_redox_ are in correspondence with the dielectric continuum function (*Z*_ox_^2^ – *Z*_red_^2^)/*r* from the Born model (eq 5) of the redox reaction entropy.
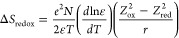
5

**Figure 5:**
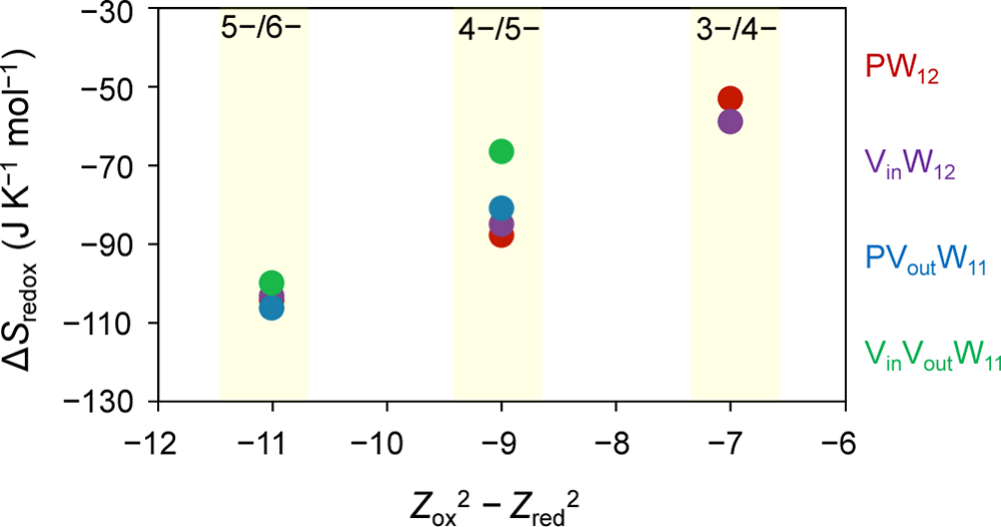
Plot of redox entropy (Δ*S*_redox_) as a function of the dielectric continuum function *Z*_ox_^2^ – *Z*_red_^2^ for the studied polyoxotungstates. The colored circles denote redox entropy values for the three charge state changes (highlighted in yellow) of respective clusters; red, purple, blue, and green circles denote data for **PW**_**12**_, **V**_**in**_**W**_**12**_, **PV**_**out**_**W**_**11**_, and **V**_**in**_**V**_**out**_**W**_**11**_ clusters, respectively.

In this equation, *Z*_ox_ and *Z*_red_ represent the charge of the oxidized and reduced cluster species, respectively, *r* denotes the effective radius of the cluster, *e* is the elementary charge, *T* is the temperature, *N* is Avogadro’s number, and ε is the static dielectric constant of the solvent.^16^ It should be noted that the radius of the aforementioned clusters does not change significantly with charge.^49,53^ This phenomenon is further supported by the experimentally derived diffusion coefficients of the studied polyoxotungstates, which are nearly equal (Tables S1 and S2). The radii of gyration for **PW**_**12**_, **V**_**in**_**W**_**12**_, **PV**_**out**_**W**_**11**_, and **V**_**in**_**V**_**out**_**W**_**11**_ were calculated using Multiwfn 3.8(dev) by adopting the crystal structures of the clusters and are provided in Table S3. Accordingly, eq 5 can be modified to eq 6 to account for the near constant radius during the electron-transfer reaction of polyoxotungstates and the obtained Δ*S*_redox_ values can be correlated to *Z*_ox_^2^ – *Z*_red_^2^ as depicted in Figure 5.
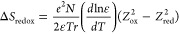
6

Our results indicate that the redox entropies of polyoxotungstates are at least partially determined by their electrostatic interactions with the surroundings. The large degree of solvent reorganization around the penta-anionic clusters as compared to tetra- and trianionic polyoxotungstates suggest that the nonspecific interactions between the Keggin polyanions and polar acetonitrile molecules and/or noncoordinating tetrabutylammonium cations increase with higher charge density. The foregoing results on the Δ*S*_redox_ values of Keggin-type POMs exhibit greater linearity with the dielectric continuum function *Z*_ox_^2^ – *Z*_red_^2^ than the Wells–Dawson ions recently reported.^49^ The experimental data published for the redox entropy of [P_2_W_18_O_62_]^6–^ suggest that there is no clear correlation between Δ*S*_redox_ and the charge state of the redox couple.^49^ For example, in their study, the reported entropy values seem to follow the order 7–/8– > 6–/7– > 10–/12– > 8–/10– for the corresponding charge states of the Wells–Dawson cluster. In contrast, the heteroatom-doped polyoxotungstates studied in this work linearly follow the Born expression, as indicated by their goodness-of-fit values (Figure S35).

While our data demonstrate a linear relation between the dielectric continuum function *Z*_ox_^2^ – *Z*_red_^2^ and redox reaction entropies in polyoxotungstate clusters, no direct correspondence between the Δ*S*_redox_ and *k*_0_ values was observed. This suggests that the kinetics of charge transfer in the studied doped polyoxotungstates is not solely governed by the structural reorganization occurring during electron transfer. We attribute this behavior to a possible compensation between the enthalpies and entropies of activation for the electroreduction processes that reflects in the net *k*_0_ values. This trait is reminiscent of the self-assembly behavior of extended POM-based materials, where the association constants are dictated by the enthalpy–entropy compensation dynamics—the process is driven enthalpically but displays an entropic penalty.^54−56^ Future work will focus on probing the enthalpic contribution to the overall Gibbs free energy by leveraging the benefits of electrochemical strategies in elucidating the rate constants of unimolecular redox reactions, which is rare in solution-phase chemical kinetics.

## Conclusions

In this study, we describe the influence of cluster charge and the properties and arrangement of heteroatom dopants on the kinetics and thermodynamics of electron transfer in Keggin-type polyoxometalates. We demonstrate that while there is minimal impact of spatial location of vanadium substitution (central vs addenda) on the electrokinetics of V-based redox events, the charge-transfer ability of W ions within the assembly is impacted by the presence of a redox-innocent vs redox-active heteroatom. Specifically, a polyoxotungstate bearing a central phosphorus heteroatom exhibits faster rates of reduction for W-based redox events as compared to V-substituted analogs. Furthermore, our variable temperature electrochemistry results illustrate that redox entropies in Keggin-type polyoxotungstates are primarily governed by electrostatics and the linear correlation between Δ*S*_redox_ and the difference in the square of the charge for the oxidized and the reduced states (*Z*_ox_^2^ – *Z*_red_^2^) follows the Born model of solvation. Overall, we anticipate that these results will motivate further avenues in understanding the kinetic and thermodynamic parameters of charge-transfer reactions in POM-based systems to enable the design of functional materials for targeted applications in energy and catalysis.

## Supporting Information Available

Sample calculation for Δ*S*_redox_, additional analytical and electrochemical data, and crystallographic parameters (PDF)
