# Supplementary material for: Implications of Charge and Heteroatom Dopants on the Thermodynamics and Kinetics of Redox Reactions in Keggin-Type Polyoxometalates
Source: ACS Mater Au. 2024 Nov 27;5(1):200–10. doi: 10.1021/acsmaterialsau.4c00136 (PMC11718532; doi:10.1021/acsmaterialsau.4c00136)
Supplement: Supplementary file 1 — mg4c00136_si_001.pdf [file mg4c00136_si_001.pdf]

Supporting Information for:

Implications of Charge and Heteroatom Dopants on the Thermodynamics and Kinetics of Redox Reactions in Keggin-Type Polyoxometalates

*Mamta Dagar, Anyesh De, Zhou Lu, Ellen M. Matson**, and *Agnes E. Thorarinsdottir**
Department of Chemistry, University of Rochester, Rochester, New York 14627, USA

*Correspondence to: matson@chem.rochester.edu (E.M.M.);
agnes.thorarinsdottir@rochester.edu (A.E.T.)

Table of Contents

A. Supplementary Text
Sample calculation for ΔS_{redox} estimation S3

B. Supplementary Figures
Figure S1. Randles–Ševčík analysis of the 3−/4− redox couple of **PW_{12}** S4
Figure S2. Randles–Ševčík analysis of the 4−/5− redox couple of **PW_{12}** S5
Figure S3. Randles–Ševčík analysis of the 5−/6− redox couple of **PW_{12}** S6
Figure S4. Randles–Ševčík analysis of the 3−/4− redox couple of **$V_{in}W_{12}$** S7
Figure S5. Randles–Ševčík analysis of the 4−/5− redox couple of **$V_{in}W_{12}$** S8
Figure S6. Randles–Ševčík analysis of the 5−/6− redox couple of **$V_{in}W_{12}$** S9
Figure S7. Nicholson analysis of the 3−/4− redox couple of **PW_{12}** S10
Figure S8. Nicholson analysis of the 4−/5− redox couple of **PW_{12}** S10
Figure S9. Nicholson analysis of the 5−/6− redox couple of **PW_{12}** S11
Figure S10. Nicholson analysis of the 3−/4− redox couple of **$V_{in}W_{12}$** S11
Figure S11. Nicholson analysis of the 4−/5− redox couple of **$V_{in}W_{12}$** S12
Figure S12. Nicholson analysis of the 5−/6− redox couple of **$V_{in}W_{12}$** S12
Figure S13. Randles–Ševčík analysis of the 4−/5− redox couple of **$PV_{out}W_{11}$** S13
Figure S14. Randles–Ševčík analysis of the 5−/6− redox couple of **$PV_{out}W_{11}$** S14
Figure S15. Nicholson analysis of the 4−/5− redox couple of **$PV_{out}W_{11}$** S15
Figure S16. Nicholson analysis of the 5−/6− redox couple of **$PV_{out}W_{11}$** S15
Figure S17. Randles–Ševčík analysis of the 4−/5− redox couple of **$V_{in}V_{out}W_{11}$** S16
Figure S18. Randles–Ševčík analysis of the 5−/6− redox couple of **$V_{in}V_{out}W_{11}$** S17
Figure S19. Nicholson analysis of the 4−/5− redox couple of **$V_{in}V_{out}W_{11}$** S18
Figure S20. Nicholson analysis of the 5−/6− redox couple of **$V_{in}V_{out}W_{11}$** S18

Figure S21. Nonisothermal VT-OCP analysis of Ag/$AgNO_3$ electrode S19
Figure S22. CVs of polyoxotungstates at ambient temperature S20
Figure S23. Isothermal VT-CV analysis of the 4−/5− redox couple of $\mathbf{V_{in}W_{12}}$ S21
Figure S24. Isothermal VT-CV analysis of the 5−/6− redox couple of $\mathbf{V_{in}W_{12}}$ S21
Figure S25. VT-CV measurement of $\mathbf{PW_{12}}$ in MeCN with (^{n}Bu$_4$N)(PF$_6$) S22
Figure S26. Isothermal VT-CV analysis of the 3−/4− redox couple of $\mathbf{PW_{12}}$ S22
Figure S27. Isothermal VT-CV analysis of the 4−/5− redox couple of $\mathbf{PW_{12}}$ S23
Figure S28. Isothermal VT-CV analysis of the 5−/6− redox couple of $\mathbf{PW_{12}}$ S23
Figure S29. VT-CV measurement of $\mathbf{PV_{out}W_{11}}$ in MeCN with (^{n}Bu$_4$N)(PF$_6$) S24
Figure S30. Isothermal VT-CV analysis of the 4−/5− redox couple of $\mathbf{PV_{out}W_{11}}$ S24
Figure S31. Isothermal VT-CV analysis of the 5−/6− redox couple of $\mathbf{PV_{out}W_{11}}$ S25
Figure S32. VT-CV measurement of $\mathbf{V_{in}V_{out}W_{11}}$ in MeCN with (^{n}Bu$_4$N)(PF$_6$) S25
Figure S33. Isothermal VT-CV analysis of the 4−/5− redox couple of $\mathbf{V_{in}V_{out}W_{11}}$ S26
Figure S34. Isothermal VT-CV analysis of the 5−/6− redox couple of $\mathbf{V_{in}V_{out}W_{11}}$ S26
Figure S35. Plot of ΔS_{redox} vs $Z_{ox}^2 - Z_{red}^2$ for $\mathbf{PW_{12}}$ and $\mathbf{V_{in}W_{12}}$ S27

C. Supplementary Tables

Table S1. Summary of cathodic diffusion coefficients of studied polyoxotungstates S28
Table S2. Summary of anodic diffusion coefficients of studied polyoxotungstates S29
Table S3. Summary of radii of gyration of studied polyoxotungstates S30

D. References S31

A. Supplementary Text

Sample Calculation for ΔS_{redox} Estimation. The redox entropy (ΔS_{redox}) can be estimated from the temperature dependence of the formal potential ($E^{0\prime}$), which can be estimated as the half-wave potential ($E_{1/2}$) owing to the similar diffusion coefficients of the oxidized and reduced polyoxotungstate species (**Tables S1** and **S2**), using eq S1:

$$\Delta S_{\text{redox}} = nF\left(\frac{dE^{0'}}{dT}\right) \tag{S1}$$

where F is Faraday's constant, n is the number of electrons involved in the corresponding redox process, and T is the temperature. However, in isothermal cell measurements, the recorded potentials are referenced to the Ag/$AgNO_3$ reference electrode potential. Thus, $E^{0\prime}$ at a given temperature is a summation of the measured formal potential ($E^{0'}_{meas}$) and E_{ref}, where E_{ref} is the potential of the Ag^+/Ag redox couple of the reference electrode. Accordingly, the redox reaction entropy can be expressed as eq S2:

$$\Delta S_{\text{redox}} = nF\left(\frac{dE^{0'}}{dT}\right) = nF\left(\frac{dE^{0'}_{meas}}{dT} + \frac{dE_{ref}}{dT}\right) \tag{S2}$$

For example, the average temperature coefficient ($dE^{0\prime}/dT$) for the 3−/4− redox couple of **$V_{in}W_{12}$** measured using the open circuit potential (OCP) method is −1.02(7) mV K^{-1} and that of Ag/$AgNO_3$ is +0.43(6) mV K^{-1}. Thus,

$$\Delta S_{redox} = 1 \times 96485 \text{ C mol}^{-1} \times (-1.02 + 0.43) \text{ mV K}^{-1}$$
$$\Delta S_{redox} = -57312.09 \text{ C mol}^{-1} \times 10^{-3} \text{ V} \times 1 \text{ K}^{-1}$$
$$\Delta S_{redox} = -57(6) \text{ J K}^{-1} \text{ mol}^{-1}$$

B. Supplementary Figures

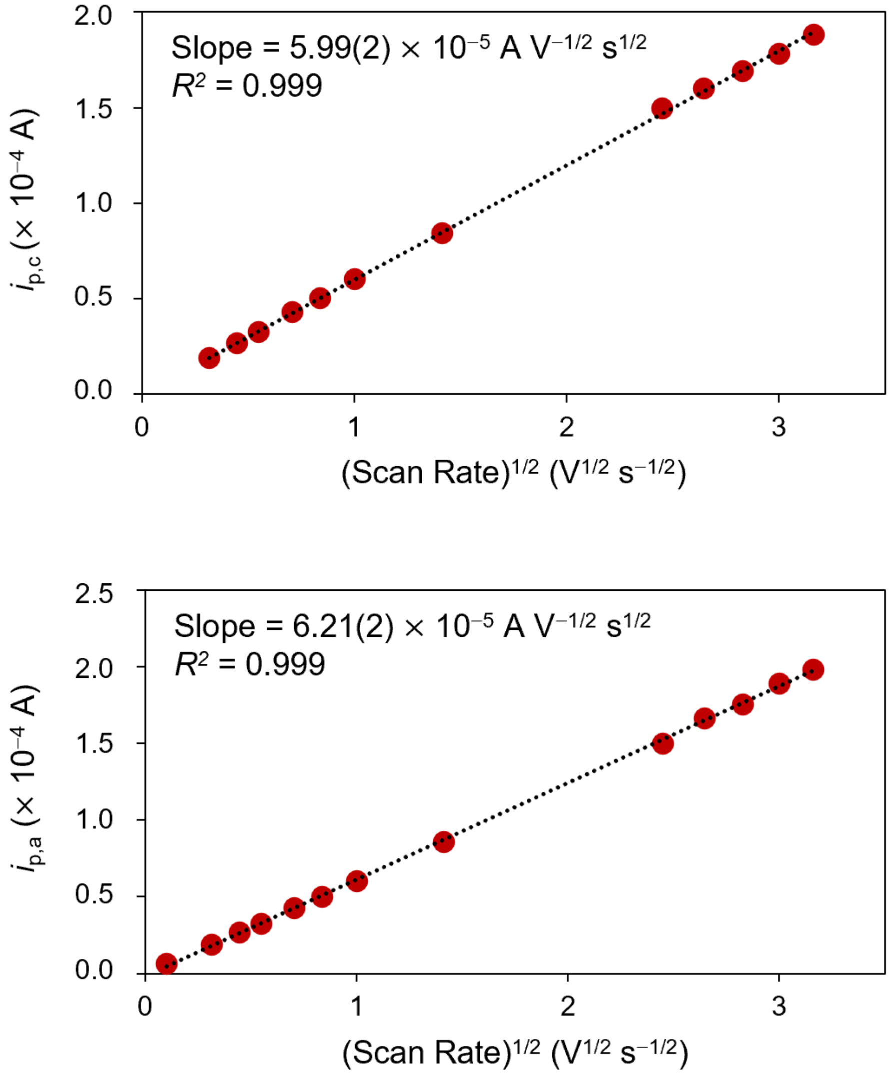

Figure S1. Randles–Ševčík analysis of the 3−/4− redox couple (cathodic wave, top; anodic wave, bottom) of **PW_{12}** in acetonitrile containing 0.1 M ($^{n}Bu_4N$)(PF_6) supporting electrolyte at ambient temperature. $i_{p,c}$ and $i_{p,a}$ denote the cathodic and anodic peak currents, respectively. The red circles denote the experimental data, and the black dotted lines represent the linear fits to the data. The error in the slope corresponds to the standard error associated with each data set in the 95% confidence interval of the regression analysis. The average diffusion coefficient (estimated using eqs 1 and 2 in the main text) obtained using the slope of the linear fit to the data for the cathodic wave was utilized to calculate the rate constant of electron transfer for the 3−/4− redox couple.

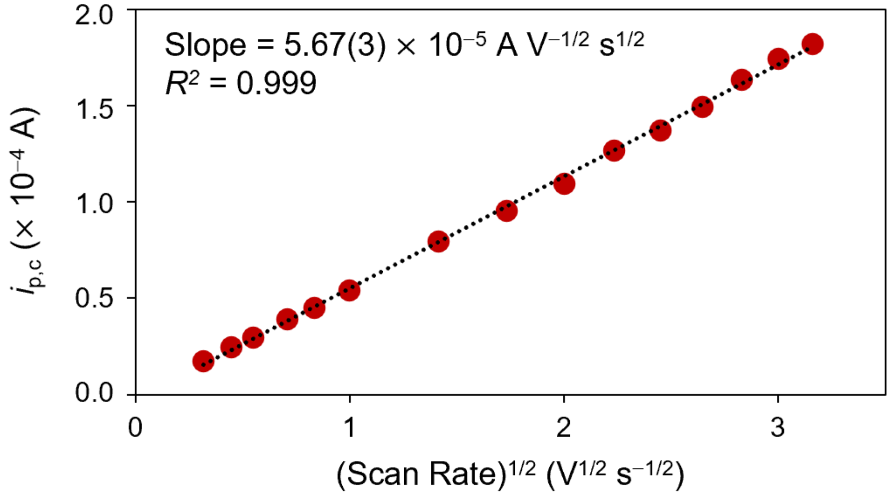

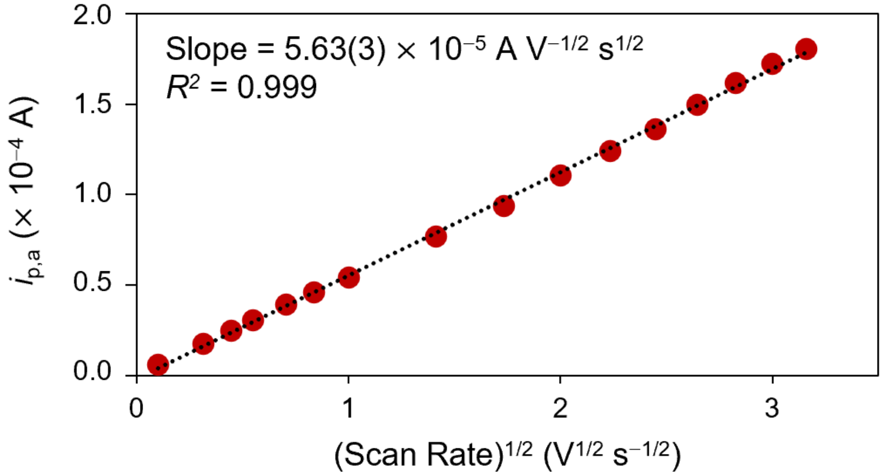

Figure S2. Randles–Ševčík analysis of the 4−/5− redox couple (cathodic wave, top; anodic wave, bottom) of **PW_{12}** in acetonitrile containing 0.1 M (^{n}Bu_4N)(PF_6) supporting electrolyte at ambient temperature. $i_{p,c}$ and $i_{p,a}$ denote the cathodic and anodic peak currents, respectively. The red circles denote the experimental data, and the black dotted lines represent the linear fits to the data. The error in the slope corresponds to the standard error associated with each data set in the 95% confidence interval of the regression analysis. The average diffusion coefficient (estimated using eqs 1 and 2 in the main text) obtained using the slope of the linear fit to the data for the cathodic wave was utilized to calculate the rate constant of electron transfer for the 4−/5− redox couple.

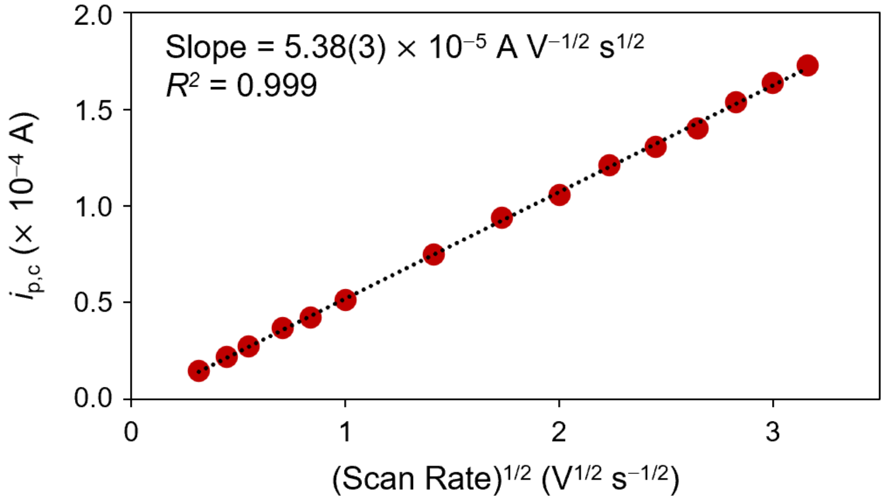

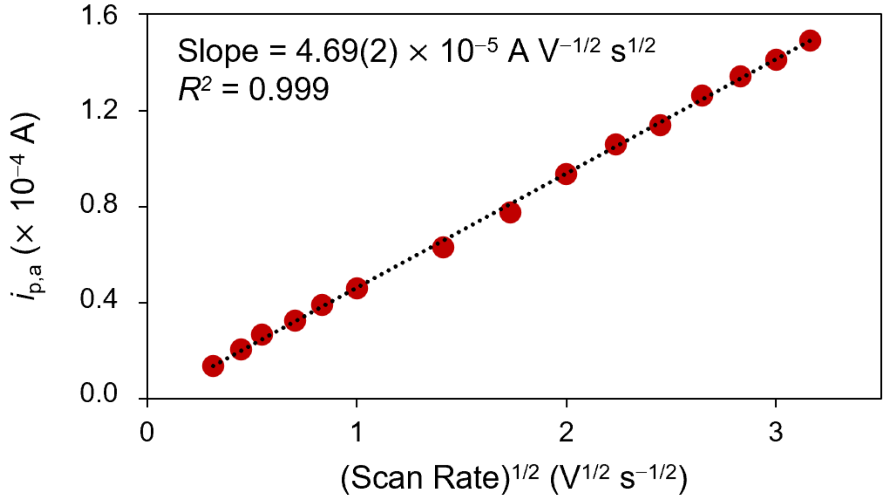

Figure S3. Randles–Ševčík analysis of the 5−/6− redox couple (cathodic wave, top; anodic wave, bottom) of **PW_{12}** in acetonitrile containing 0.1 M ($^{n}Bu_4N$)(PF_6) supporting electrolyte at ambient temperature. $i_{p,c}$ and $i_{p,a}$ denote the cathodic and anodic peak currents, respectively. The red circles denote the experimental data, and the black dotted lines represent the linear fits to the data. The error in the slope corresponds to the standard error associated with each data set in the 95% confidence interval of the regression analysis. The average diffusion coefficient (estimated using eqs 1 and 2 in the main text) obtained using the slope of the linear fit to the data for the cathodic wave was utilized to calculate the rate constant of electron transfer for the 5−/6− redox couple.

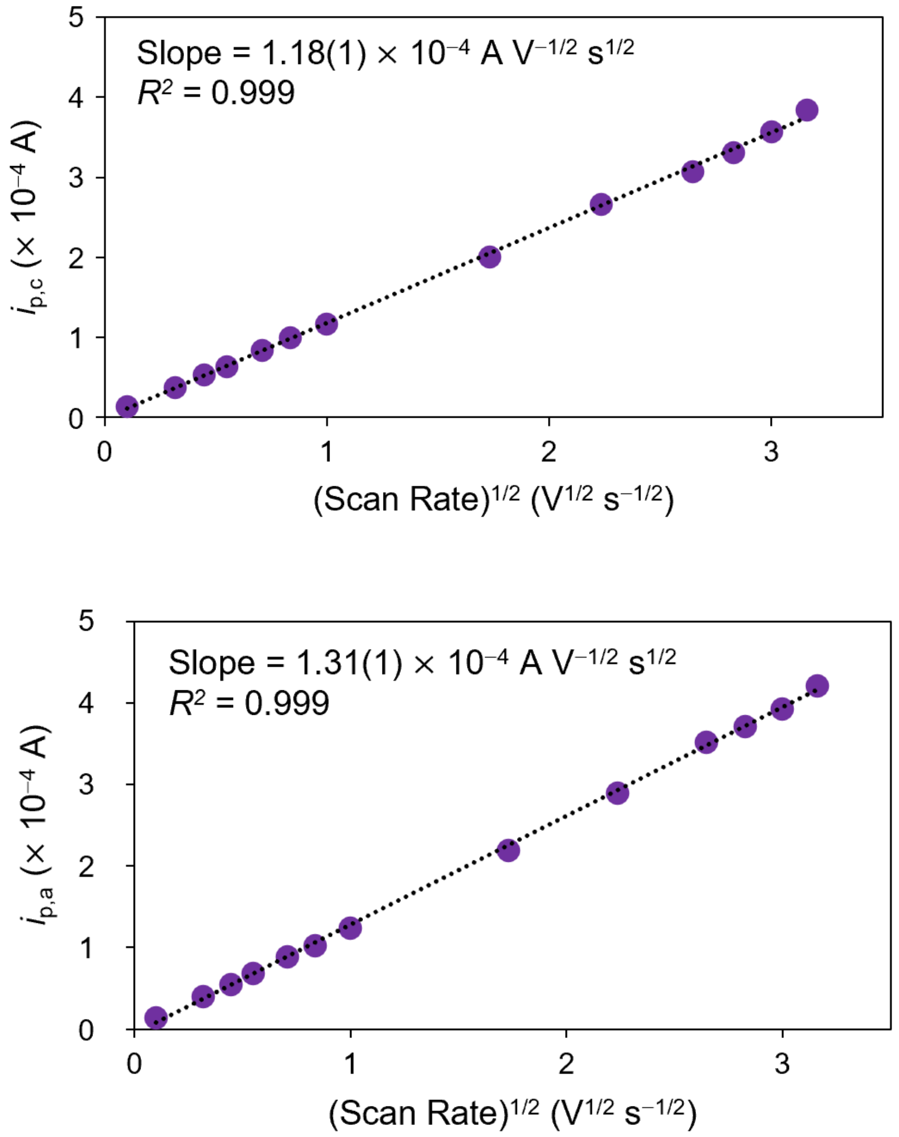

Figure S4. Randles–Ševčík analysis of the 3−/4− redox couple (cathodic wave, top; anodic wave, bottom) of **$V_{in}W_{12}$** in acetonitrile containing 0.1 M (^{n}Bu$_4$N)(PF$_6$) supporting electrolyte at ambient temperature. $i_{p,c}$ and $i_{p,a}$ denote the cathodic and anodic peak currents, respectively. The purple circles denote the experimental data, and the black dotted lines represent the linear fits to the data. The error in the slope corresponds to the standard error associated with each data set in the 95% confidence interval of the regression analysis. The average diffusion coefficient (estimated using eqs 1 and 2 in the main text) obtained using the slope of the linear fit to the data for the cathodic wave was utilized to calculate the rate constant of electron transfer for the 3−/4− redox couple.

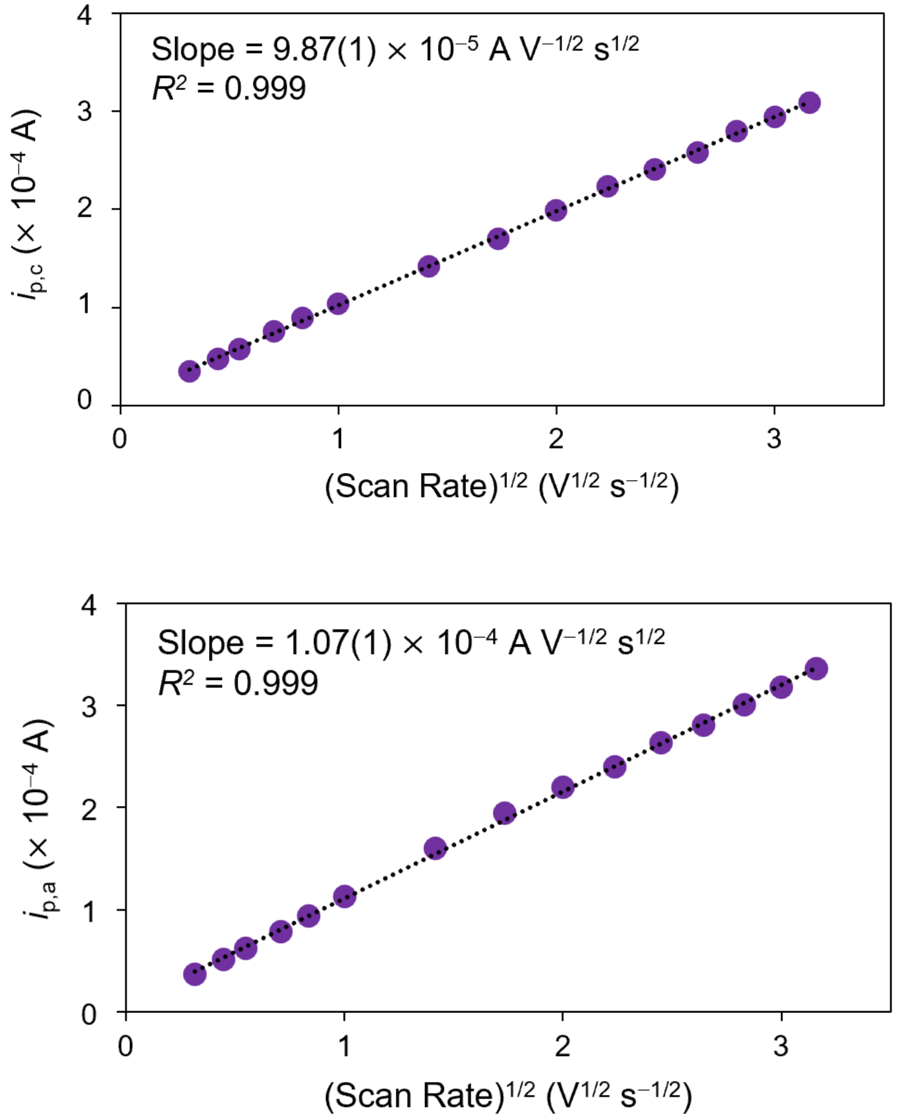

Figure S5. Randles–Ševčík analysis of the 4−/5− redox couple (cathodic wave, top; anodic wave, bottom) of $\mathbf{V_{in}W_{12}}$ in acetonitrile containing 0.1 M (^{n}Bu$_4$N)(PF$_6$) supporting electrolyte at ambient temperature. $i_{p,c}$ and $i_{p,a}$ denote the cathodic and anodic peak currents, respectively. The purple circles denote the experimental data, and the black dotted lines represent the linear fits to the data. The error in the slope corresponds to the standard error associated with each data set in the 95% confidence interval of the regression analysis. The average diffusion coefficient (estimated using eqs 1 and 2 in the main text) obtained using the slope of the linear fit to the data for the cathodic wave was utilized to calculate the rate constant of electron transfer for the 4−/5− redox couple.

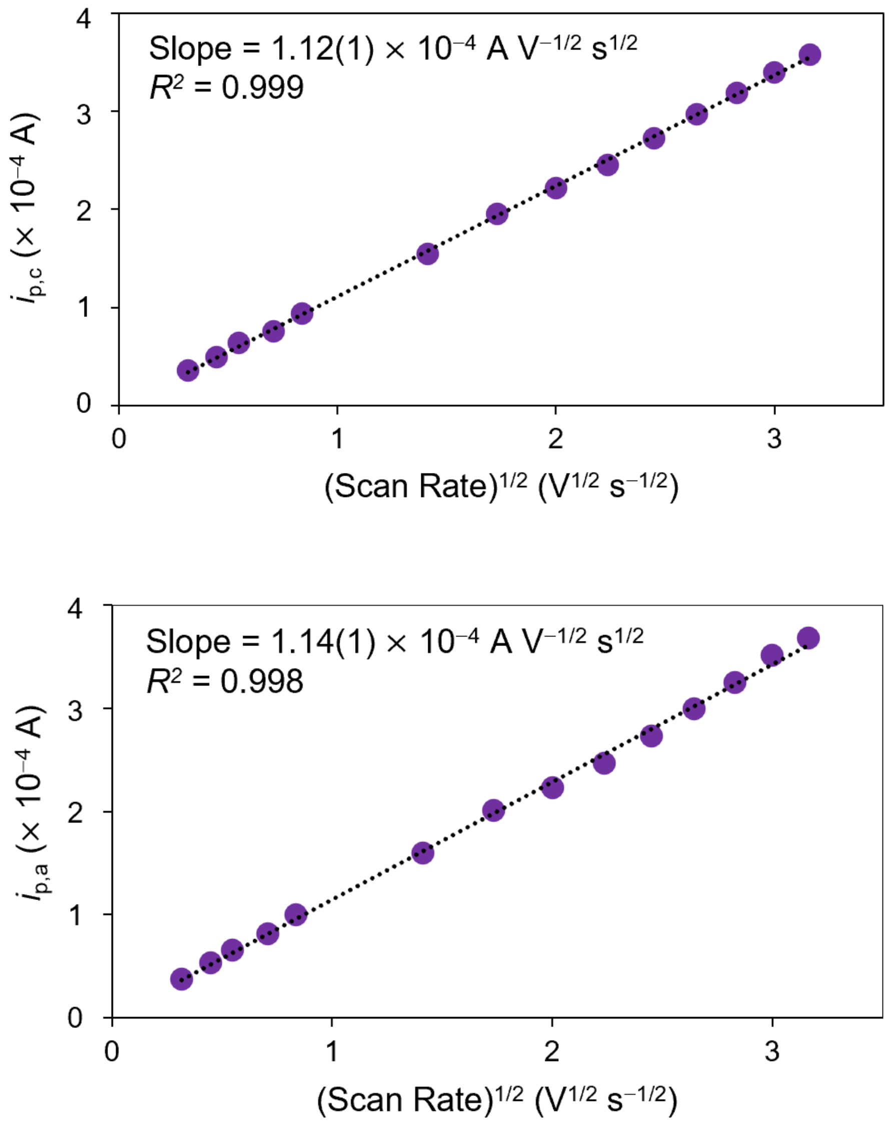

Figure S6. Randles–Ševčík analysis of the 5−/6− redox couple (cathodic wave, top; anodic wave, bottom) of **$V_{in}W_{12}$** in acetonitrile containing 0.1 M (^{n}Bu_4N)(PF_6) supporting electrolyte at ambient temperature. $i_{p,c}$ and $i_{p,a}$ denote the cathodic and anodic peak currents, respectively. The purple circles denote the experimental data, and the black dotted lines represent the linear fits to the data. The error in the slope corresponds to the standard error associated with each data set in the 95% confidence interval of the regression analysis. The average diffusion coefficient (estimated using eqs 1 and 2 in the main text) obtained using the slope of the linear fit to the data for the cathodic wave was utilized to calculate the rate constant of electron transfer for the 5−/6− redox couple.

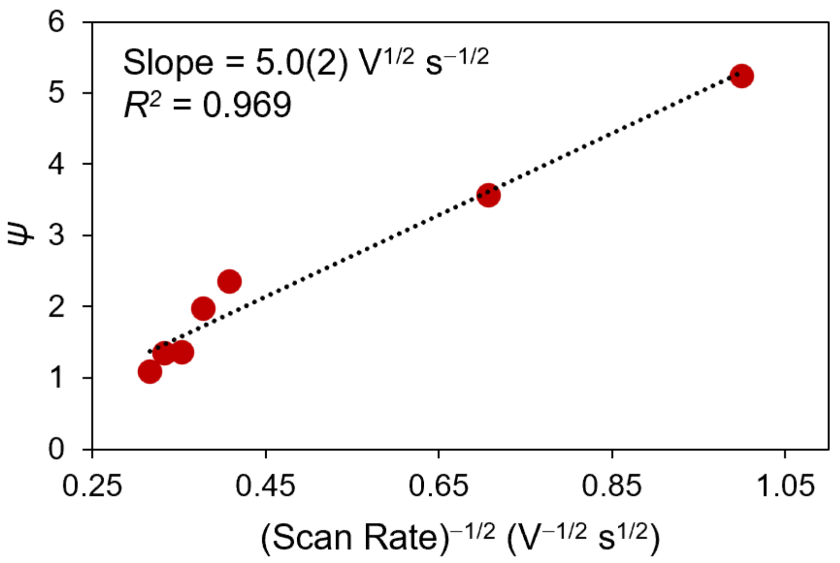

Figure S7. Plot of ψ vs inverse square root of scan rate for the 3−/4− redox couple of $\mathbf{PW_{12}}$ obtained from variable scan rate cyclic voltammetry (CV) data collected in acetonitrile containing 0.1 M (^{n}Bu$_4$N)(PF$_6$) supporting electrolyte at ambient temperature. The red circles denote the experimental data, and the black dotted line represents the linear fit to the data. The error in the slope corresponds to the standard error associated with each data set in the 95% confidence interval of the regression analysis.

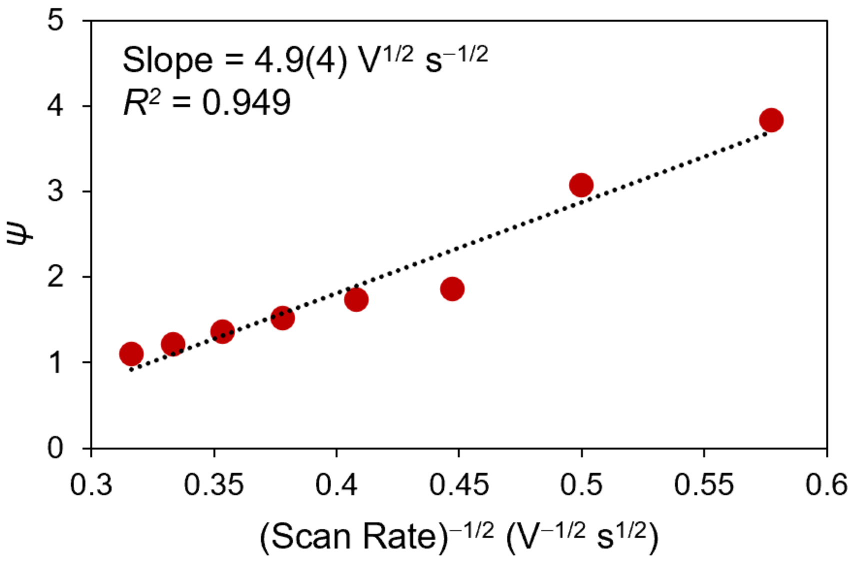

Figure S8. Plot of ψ vs inverse square root of scan rate for the 4−/5− redox couple of $\mathbf{PW_{12}}$ obtained from variable scan rate CV data collected in acetonitrile containing 0.1 M (^{n}Bu$_4$N)(PF$_6$) supporting electrolyte at ambient temperature. The red circles denote the experimental data, and the black dotted line represents the linear fit to the data. The error in the slope corresponds to the standard error associated with each data set in the 95% confidence interval of the regression analysis.

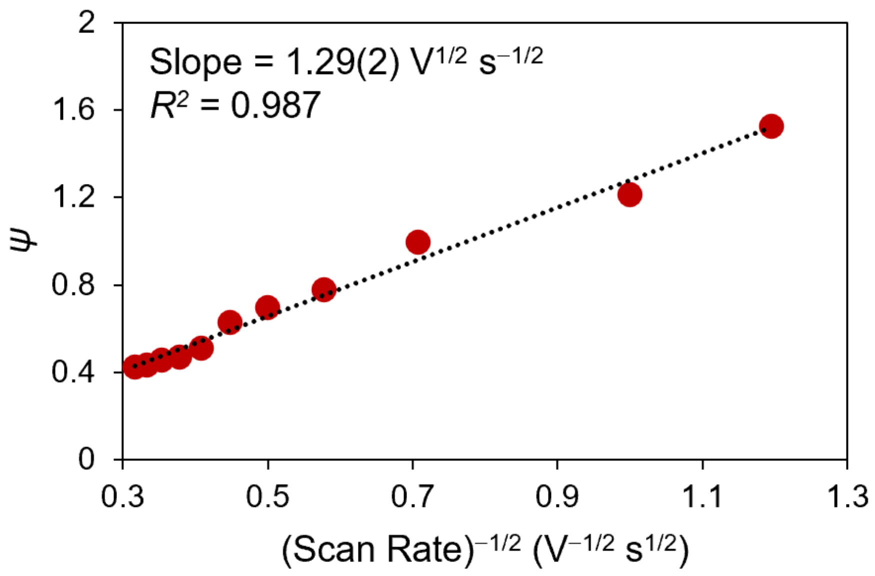

Figure S9. Plot of ψ vs inverse square root of scan rate for the 5−/6− redox couple of $\mathbf{PW_{12}}$ obtained from variable scan rate CV data collected in acetonitrile containing 0.1 M (^{n}Bu$_4$N)(PF$_6$) supporting electrolyte at ambient temperature. The red circles denote the experimental data, and the black dotted line represents the linear fit to the data. The error in the slope corresponds to the standard error associated with each data set in the 95% confidence interval of the regression analysis.

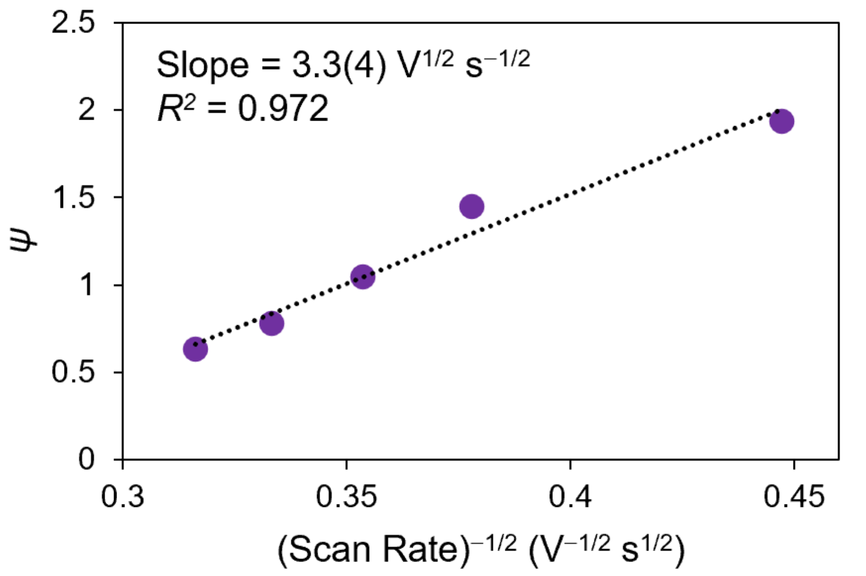

Figure S10. Plot of ψ vs inverse square root of scan rate for the 3−/4− redox couple of $\mathbf{V_{in}W_{12}}$ obtained from variable scan rate CV data collected in acetonitrile containing 0.1 M (^{n}Bu$_4$N)(PF$_6$) supporting electrolyte at ambient temperature. The purple circles denote the experimental data, and the black dotted line represents the linear fit to the data. The error in the slope corresponds to the standard error associated with each data set in the 95% confidence interval of the regression analysis.

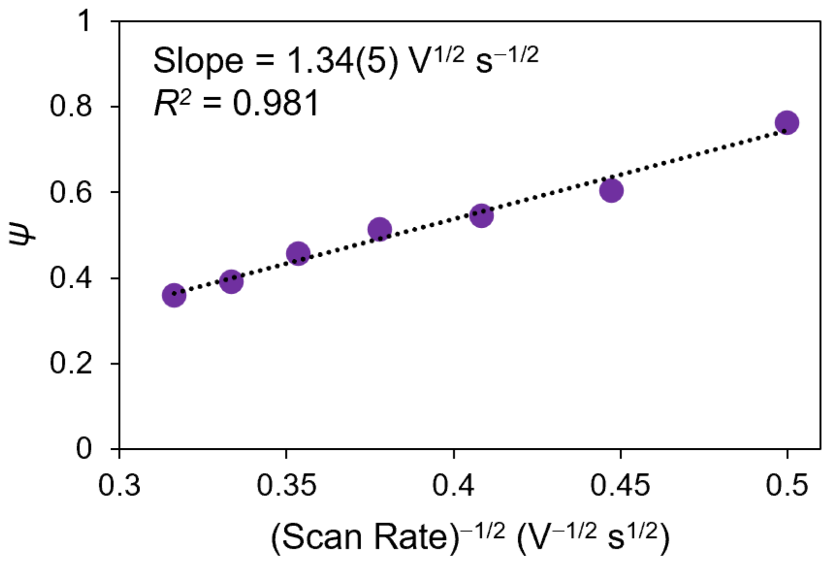

Figure S11. Plot of ψ vs inverse square root of scan rate for the 4−/5− redox couple of $\mathbf{V_{in}W_{12}}$ obtained from variable scan rate CV data collected in acetonitrile containing 0.1 M (^{n}Bu$_4$N)(PF$_6$) supporting electrolyte at ambient temperature. The purple circles denote the experimental data, and the black dotted line represents the linear fit to the data. The error in the slope corresponds to the standard error associated with each data set in the 95% confidence interval of the regression analysis.

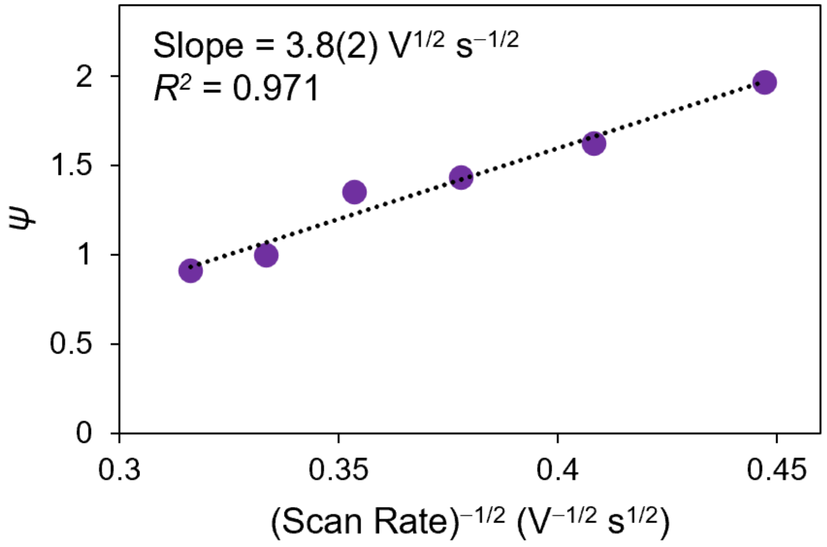

Figure S12. Plot of ψ vs inverse square root of scan rate for the 5−/6− redox couple of $\mathbf{V_{in}W_{12}}$ obtained from variable scan rate CV data collected in acetonitrile containing 0.1 M (^{n}Bu$_4$N)(PF$_6$) supporting electrolyte at ambient temperature. The purple circles denote the experimental data, and the black dotted line represents the linear fit to the data. The error in the slope corresponds to the standard error associated with each data set in the 95% confidence interval of the regression analysis.

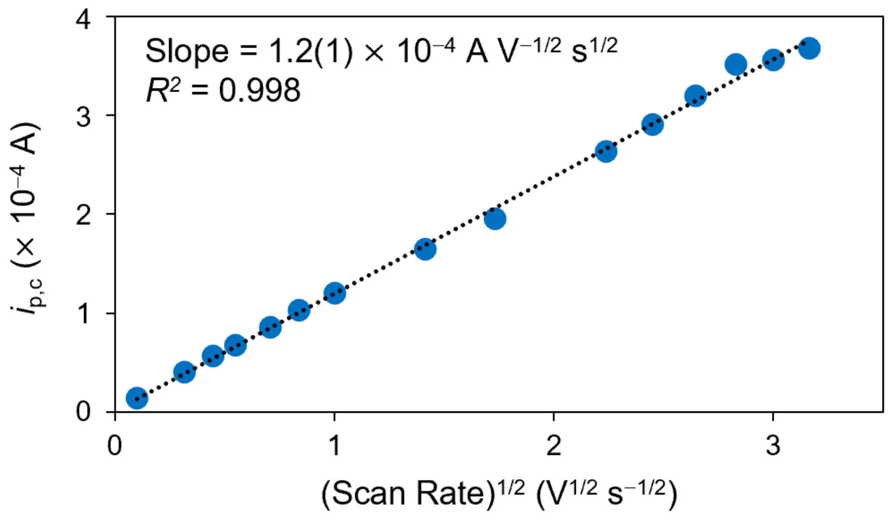

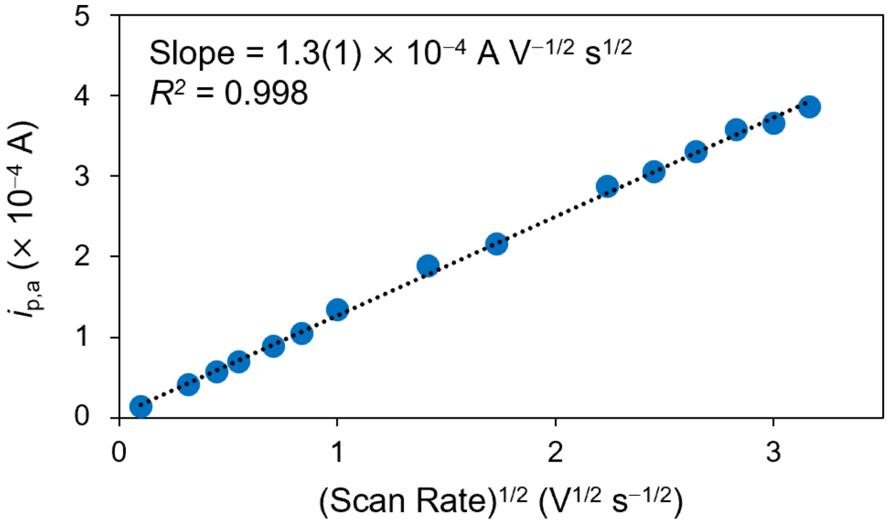

Figure S13. Randles–Ševčík analysis of the 4−/5− redox couple (cathodic wave, top; anodic wave, bottom) of **$PV_{out}W_{11}$** in acetonitrile containing 0.1 M (^{n}Bu$_4$N)(PF$_6$) supporting electrolyte at ambient temperature. $i_{p,c}$ and $i_{p,a}$ denote the cathodic and anodic peak currents, respectively. The blue circles denote the experimental data, and the black dotted lines represent the linear fits to the data. The error in the slope corresponds to the standard error associated with each data set in the 95% confidence interval of the regression analysis. The average diffusion coefficient (estimated using eqs 1 and 2 in the main text) obtained using the slope of the linear fit to the data for the cathodic wave was utilized to calculate the rate constant of electron transfer for the 4−/5− redox couple.

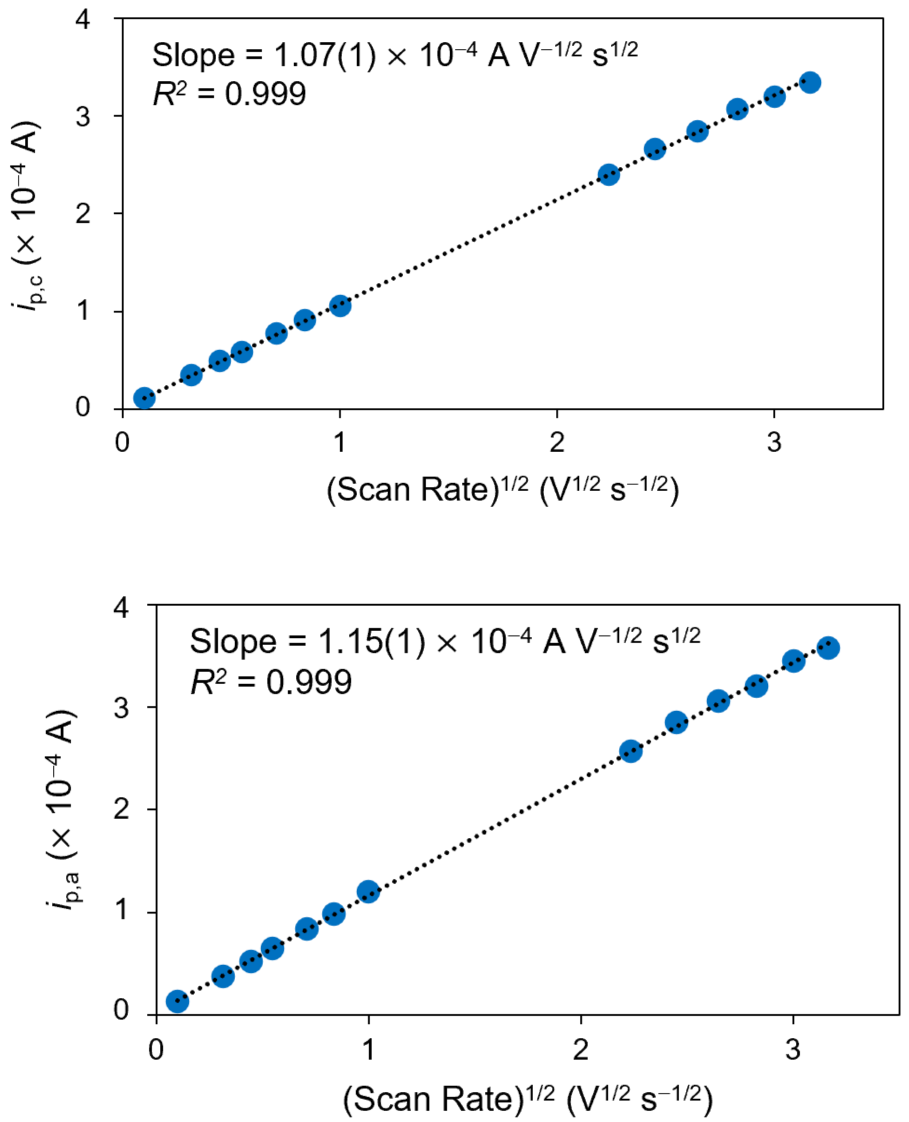

Figure S14. Randles–Ševčík analysis of the 5−/6− redox couple (cathodic wave, top; anodic wave, bottom) of **PV$_{out}$W$_{11}$** in acetonitrile containing 0.1 M (^{n}Bu$_4$N)(PF$_6$) supporting electrolyte at ambient temperature. $i_{p,c}$ and $i_{p,a}$ denote the cathodic and anodic peak currents, respectively. The blue circles denote the experimental data, and the black dotted lines represent the linear fits to the data. The error in the slope corresponds to the standard error associated with each data set in the 95% confidence interval of the regression analysis. The average diffusion coefficient (estimated using eqs 1 and 2 in the main text) obtained using the slope of the linear fit to the data for the cathodic wave was utilized to calculate the rate constant of electron transfer for the 5−/6− redox couple.

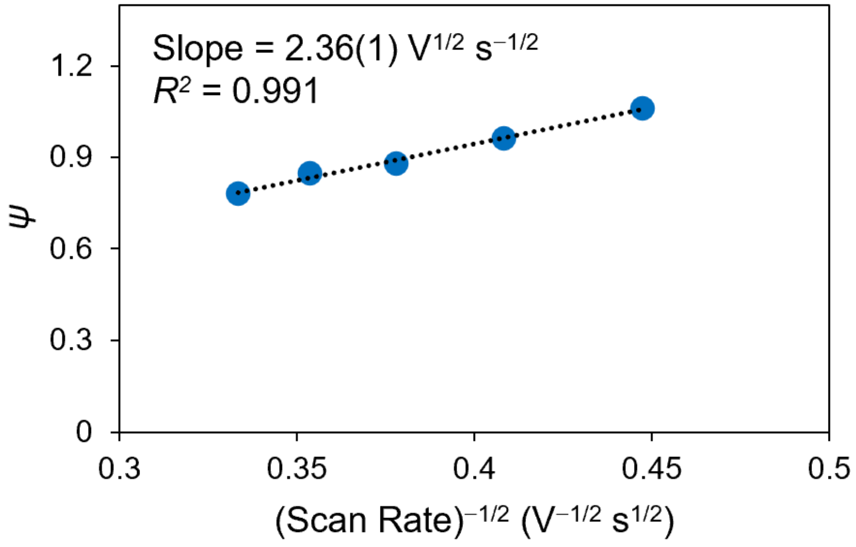

Figure S15. Plot of ψ vs inverse square root of scan rate for the 4−/5− redox couple of **PV$_{out}$W$_{11}$** obtained from variable scan rate CV data collected in acetonitrile containing 0.1 M (^{n}Bu$_4$N)(PF$_6$) supporting electrolyte at ambient temperature. The blue circles denote the experimental data, and the black dotted line represents the linear fit to the data. The error in the slope corresponds to the standard error associated with each data set in the 95% confidence interval of the regression analysis.

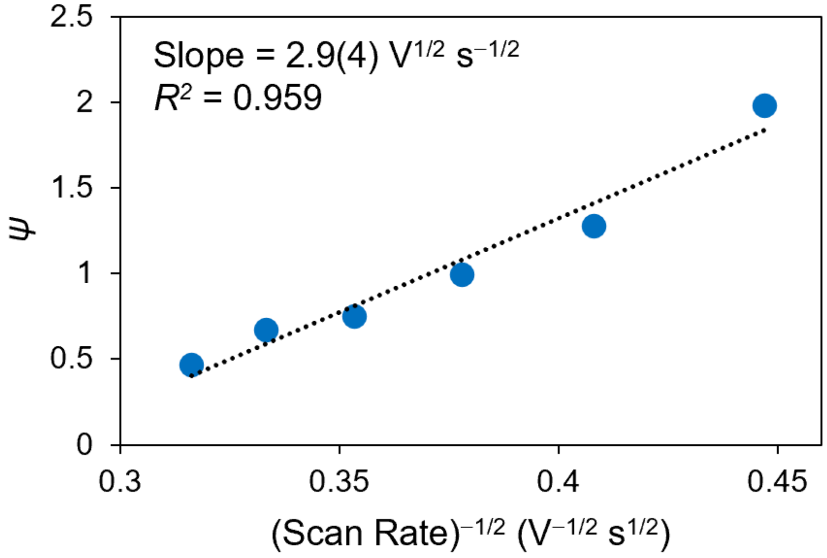

Figure S16. Plot of ψ vs inverse square root of scan rate for the 5−/6− redox couple of **PV$_{out}$W$_{11}$** obtained from variable scan rate CV data collected in acetonitrile containing 0.1 M (^{n}Bu$_4$N)(PF$_6$) supporting electrolyte at ambient temperature. The blue circles denote the experimental data, and the black dotted line represents the linear fit to the data. The error in the slope corresponds to the standard error associated with each data set in the 95% confidence interval of the regression analysis.

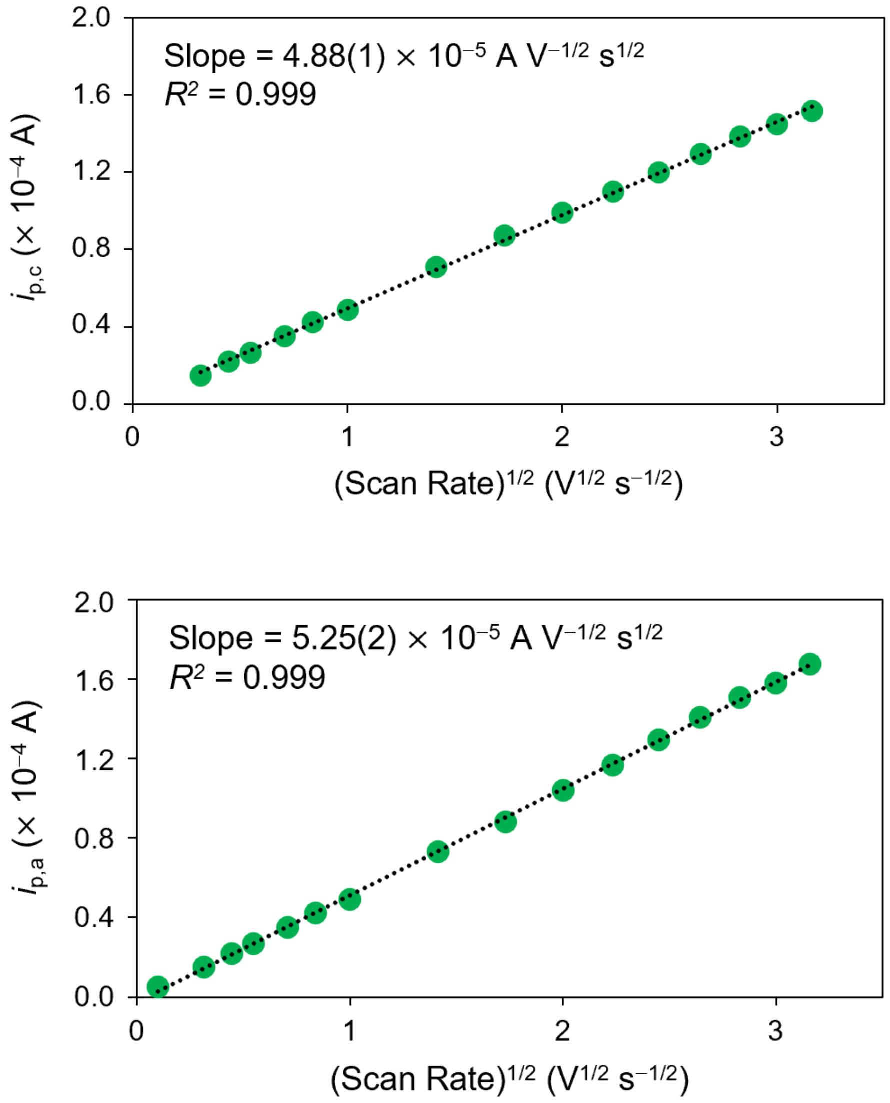

Figure S17. Randles–Ševčík analysis of the 4−/5− redox couple (cathodic wave, top; anodic wave, bottom) of $\mathbf{V_{in}V_{out}W_{11}}$ in acetonitrile containing 0.1 M (^{n}Bu$_4$N)(PF$_6$) supporting electrolyte at ambient temperature. $i_{p,c}$ and $i_{p,a}$ denote the cathodic and anodic peak currents, respectively. The green circles denote the experimental data, and the black dotted lines represent the linear fits to the data. The error in the slope corresponds to the standard error associated with each data set in the 95% confidence interval of the regression analysis. The average diffusion coefficient (estimated using eqs 1 and 2 in the main text) obtained using the slope of the linear fit to the data for the cathodic wave was utilized to calculate the rate constant of electron transfer for the 4−/5− redox couple.

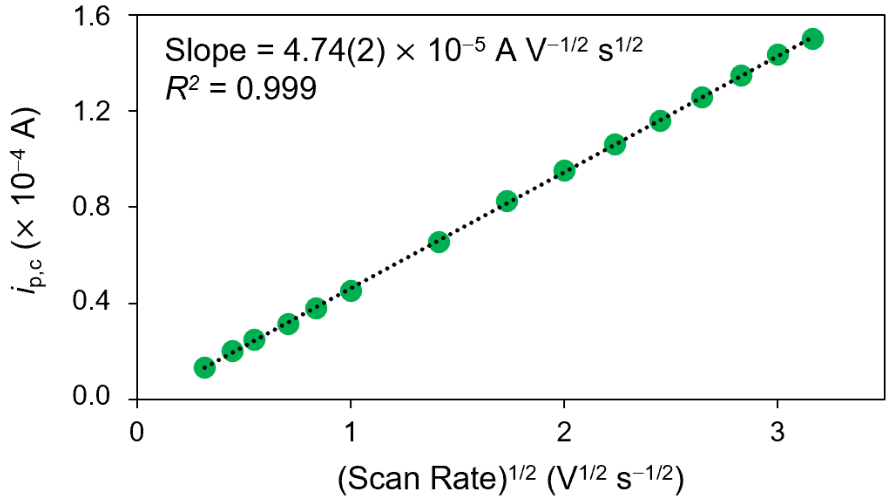

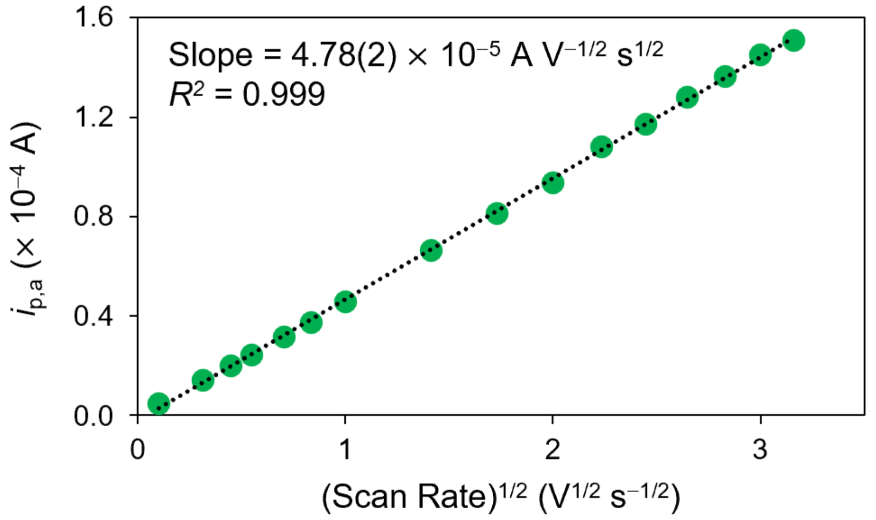

Figure S18. Randles–Ševčík analysis of the 5−/6− redox couple (cathodic wave, top; anodic wave, bottom) of $\mathbf{V_{in}V_{out}W_{11}}$ in acetonitrile containing 0.1 M (^{n}Bu$_4$N)(PF$_6$) supporting electrolyte at ambient temperature. $i_{p,c}$ and $i_{p,a}$ denote the cathodic and anodic peak currents, respectively. The green circles denote the experimental data, and the black dotted lines represent the linear fits to the data. The error in the slope corresponds to the standard error associated with each data set in the 95% confidence interval of the regression analysis. The average diffusion coefficient (estimated using eqs 1 and 2 in the main text) obtained using the slope of the linear fit to the data for the cathodic wave was utilized to calculate the rate constant of electron transfer for the 5−/6− redox couple.

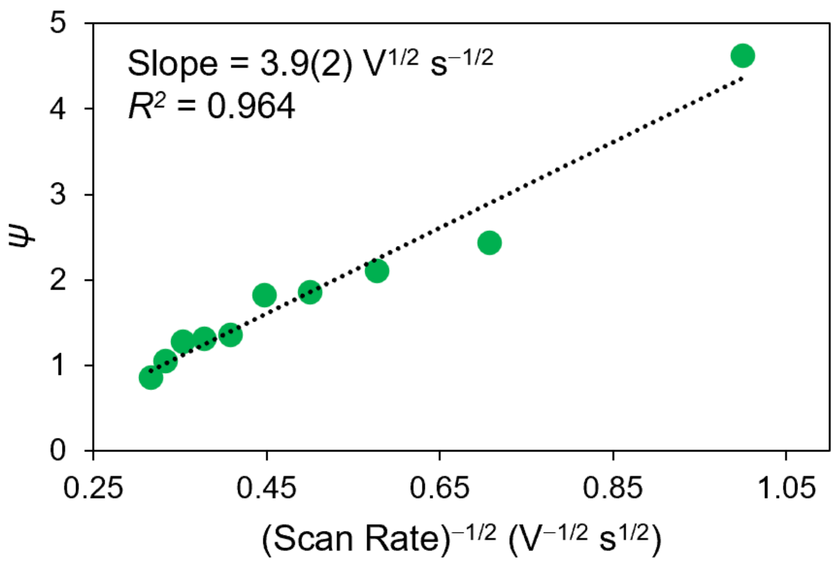

Figure S19. Plot of ψ vs inverse square root of scan rate for the 4−/5− redox couple of $\mathbf{V_{in}V_{out}W_{11}}$ obtained from variable scan rate CV data collected in acetonitrile containing 0.1 M ($^{n}Bu_4N$)(PF_6) supporting electrolyte at ambient temperature. The green circles denote the experimental data, and the black dotted line represents the linear fit to the data. The error in the slope corresponds to the standard error associated with each data set in the 95% confidence interval of the regression analysis.

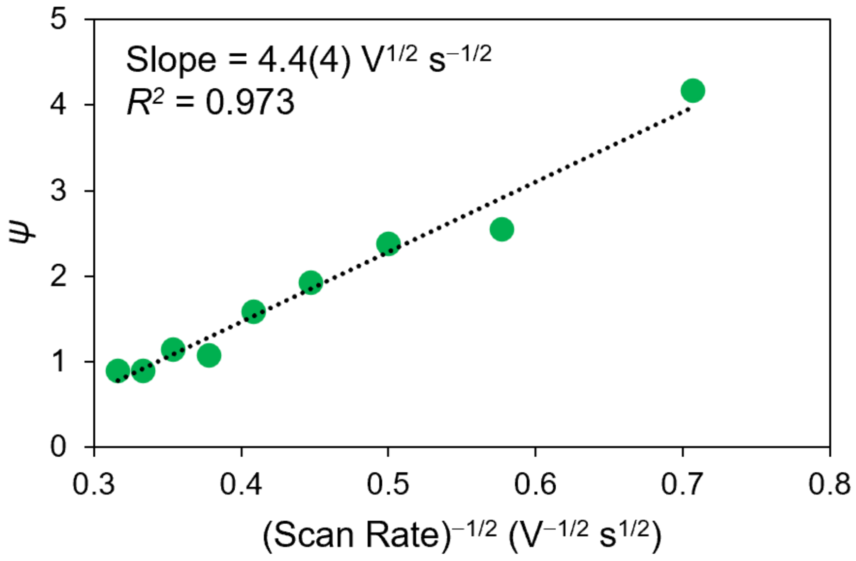

Figure S20. Plot of ψ vs inverse square root of scan rate for the 5−/6− redox couple of $\mathbf{V_{in}V_{out}W_{11}}$ obtained from variable scan rate CV data collected in acetonitrile containing 0.1 M ($^{n}Bu_4N$)(PF_6) supporting electrolyte at ambient temperature. The green circles denote the experimental data, and the black dotted line represents the linear fit to the data. The error in the slope corresponds to the standard error associated with each data set in the 95% confidence interval of the regression analysis.

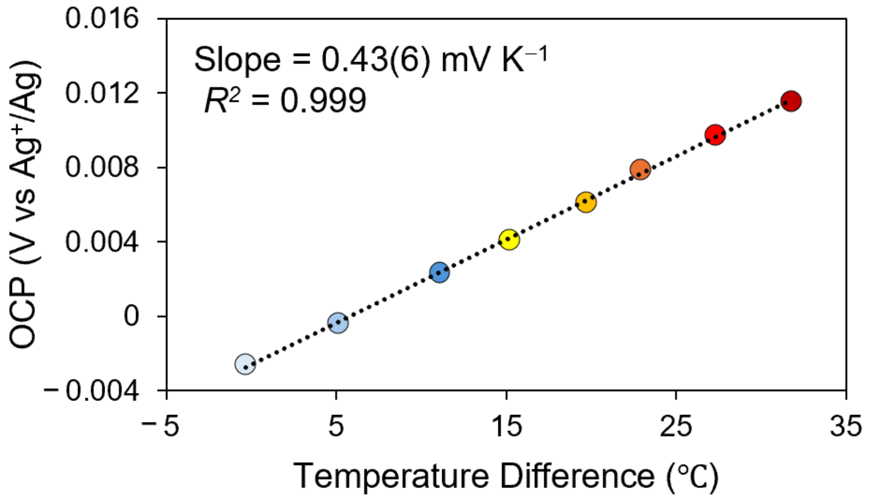

Figure S21. Representative example of the observed temperature dependence of the OCP values at steady state for Ag/$AgNO_3$ reference electrode, recorded in acetonitrile containing 0.1 M (^{n}Bu_4N)(PF_6) supporting electrolyte using nonisothermal electrochemical setup with the hot compartment heated from ~25 °C to ~45 °C. The colored circles denote the experimental data, and the black dotted line represents the linear fit to the data. The reported slope (in values of mV K^{-1}, as is the convention in the field) is an average of three independent measurements and the error in the slope corresponds to the standard deviation obtained from these measurements.

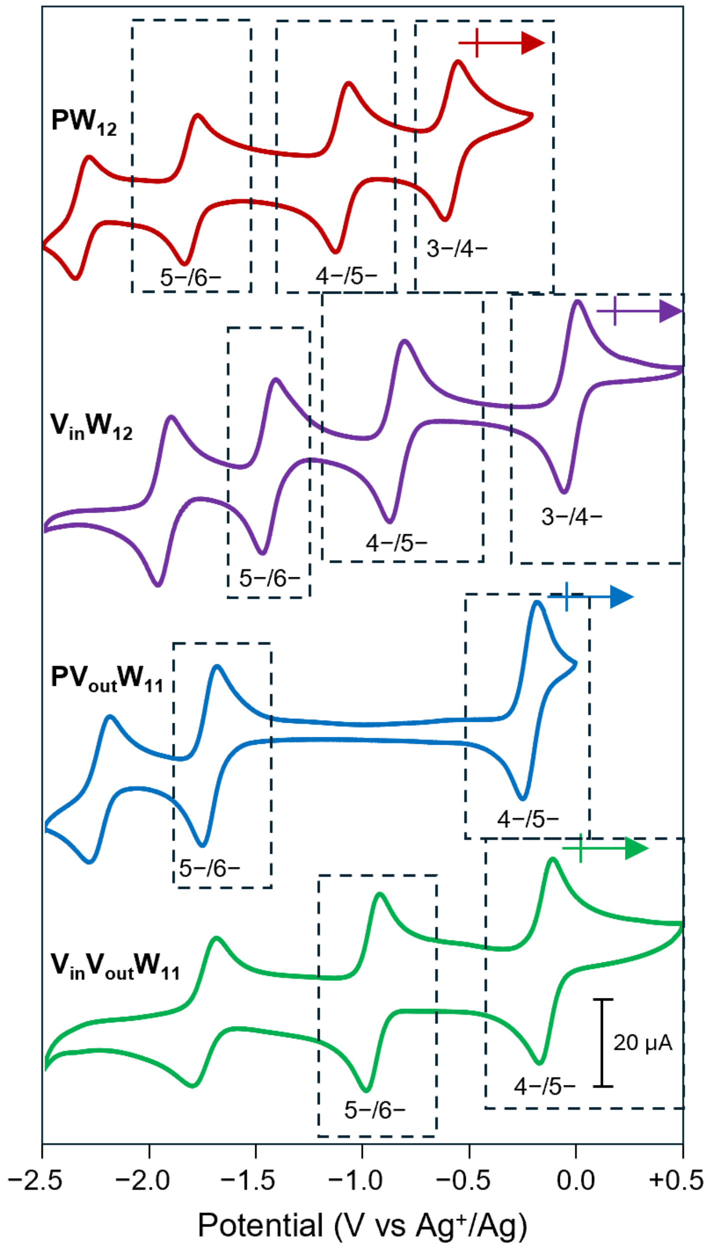

Figure S22. CVs of the studied polyoxotungstates: **PW_{12}** (red), **$V_{in}W_{12}$** (purple), **$PV_{out}W_{11}$** (blue), and **$V_{in}V_{out}W_{11}$** (green) recorded in acetonitrile containing 0.1 M ($^{n}Bu_4N$)(PF_6) supporting electrolyte at ambient temperature using a scan rate of 100 mV s^{-1}. The peaks marked in black dotted squares in the respective CVs were used for evaluating the temperature coefficients of the formal potentials. The remaining peaks were deemed unsuitable for further analysis due to the unstable nature of the respective peaks during variable temperature CV (VT-CV) measurements. The negative numbers below the voltammograms denote the charge states of the clusters corresponding to each redox wave. For VT-CV data, see **Figure S25**, **Figure 4a**, **Figure S29**, and **Figure S32** for **PW_{12}**, **$V_{in}W_{12}$**, **$PV_{out}W_{11}$**, and **$V_{in}V_{out}W_{11}$**, respectively.

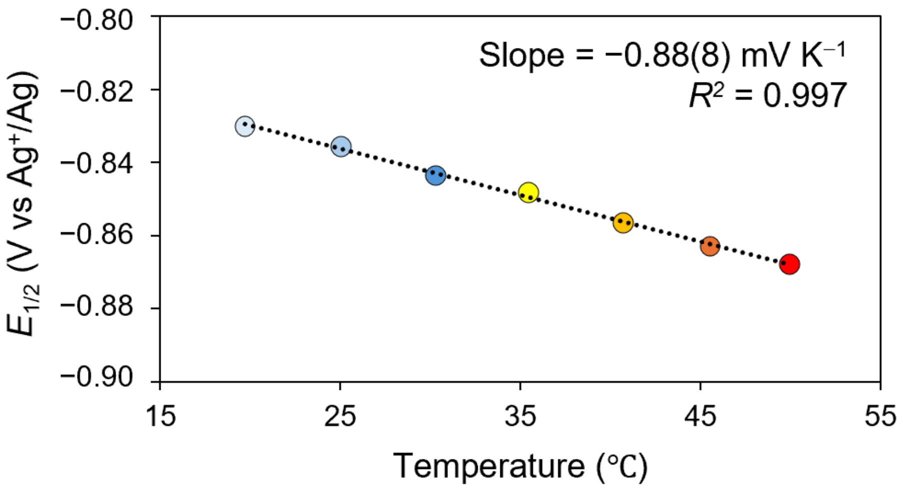

Figure S23. Representative example of the observed temperature dependence of the $E_{1/2}$ values obtained from the corresponding CVs for the 4−/5− redox couple of **V$_{\mathbf{in}}$W$_{\mathbf{12}}$** similar to those shown in **Figure 4**. The temperature for this representative data set ranges from a low temperature of 19.7 °C (blue) to a high temperature of 50.0 °C (red). The colored circles denote the experimental data, and the black dotted line represents the linear fit to the data. The reported slope (in values of mV K^{-1}, as is the convention in the field) is an average of three independent measurements and the error in the slope corresponds to the standard deviation obtained from these measurements.

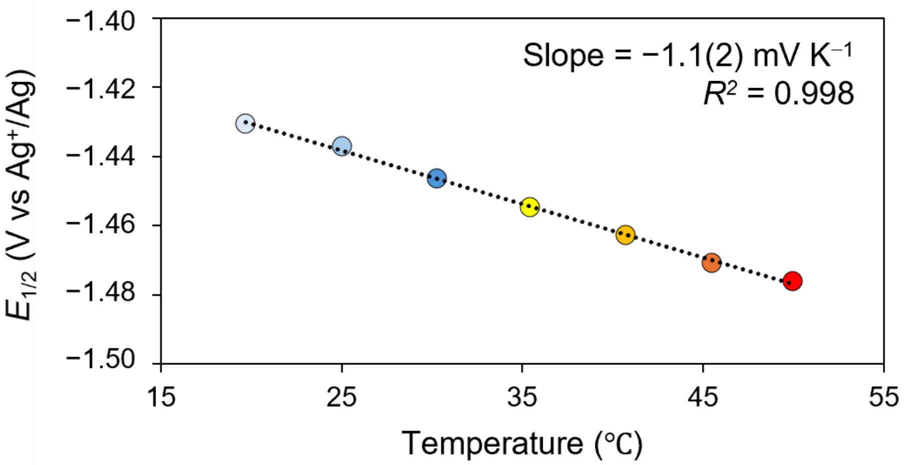

Figure S24. Representative example of the observed temperature dependence of the $E_{1/2}$ values obtained from the corresponding CVs for the 5−/6− redox couple of **V$_{\mathbf{in}}$W$_{\mathbf{12}}$** similar to those shown in **Figure 4**. The temperature for this representative data set ranges from a low temperature of 19.7 °C (blue) to a high temperature of 50.0 °C (red). The colored circles denote the experimental data, and the black dotted line represents the linear fit to the data. The reported slope (in values of mV K^{-1}, as is the convention in the field) is an average of three independent measurements and the error in the slope corresponds to the standard deviation obtained from these measurements.

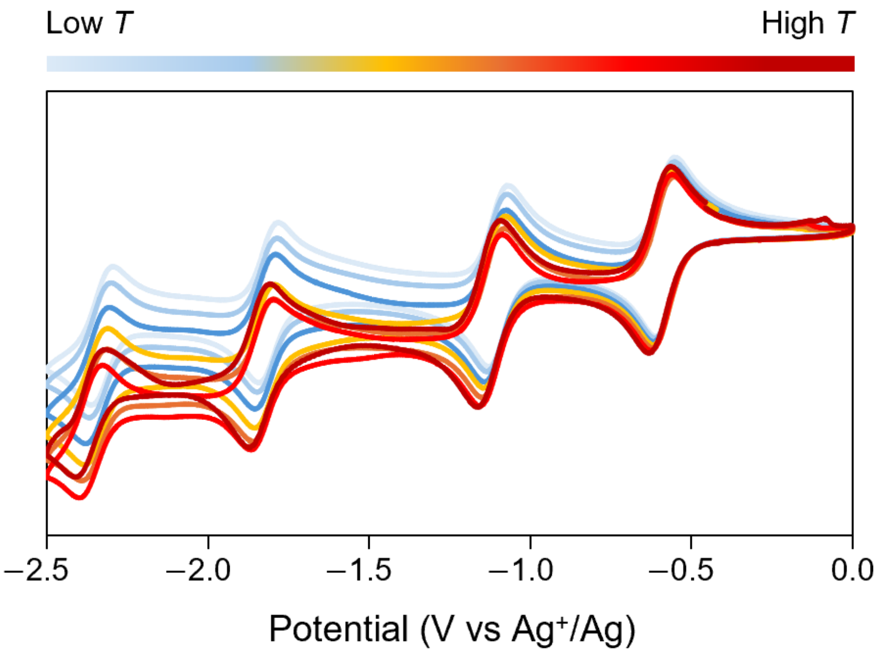

Figure S25. VT-CV data of 1 mM solution of **PW_{12}** in acetonitrile containing 0.1 M ($^{n}Bu_4N$)(PF_6) supporting electrolyte, collected at a scan rate of 100 mV s^{-1} using isothermal electrochemical setup from a low temperature of 24.4 °C (blue) to a high temperature of 54.5 °C (red). See the corresponding plots of $E_{1/2}$ vs temperature in **Figures S26–S28**.

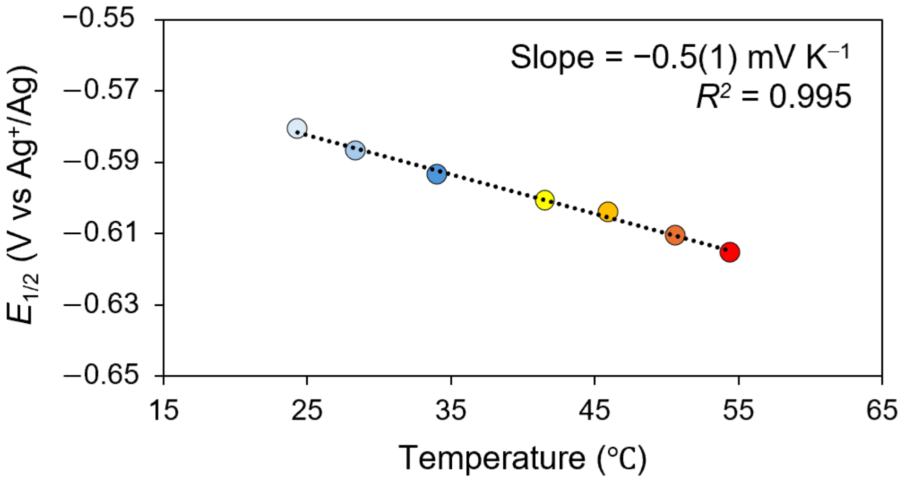

Figure S26. Representative example of the observed temperature dependence of the $E_{1/2}$ values obtained from the corresponding CVs for the 3−/4− redox couple of **PW_{12}** as shown in **Figure S25**. The colored circles denote the experimental data, and the black dotted line represents the linear fit to the data. The reported slope (in values of mV K^{-1}, as is the convention in the field) is an average of three independent measurements and the error in the slope corresponds to the standard deviation obtained from these measurements.

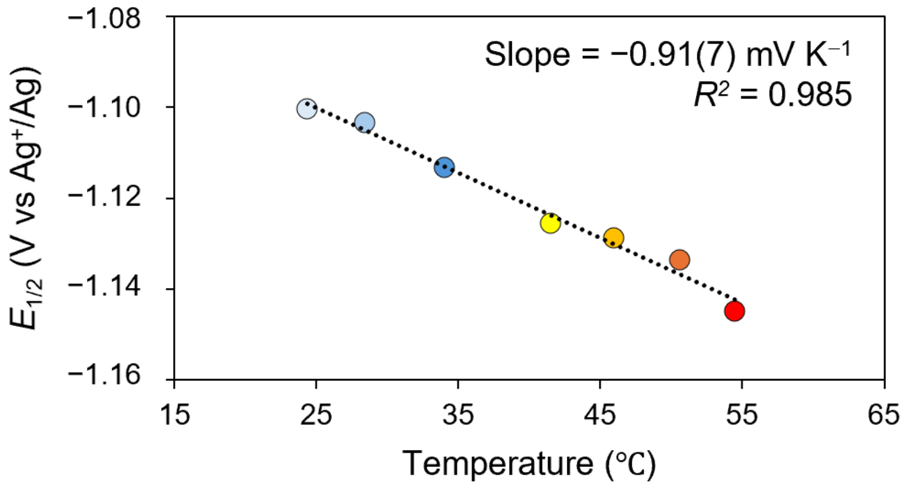

Figure S27. Representative example of the observed temperature dependence of the $E_{1/2}$ values obtained from the corresponding CVs for the 4−/5− redox couple of **PW$_{12}$** as shown in **Figure S25**. The colored circles denote the experimental data, and the black dotted line represents the linear fit to the data. The reported slope (in values of mV K^{-1}, as is the convention in the field) is an average of three independent measurements and the error in the slope corresponds to the standard deviation obtained from these measurements.

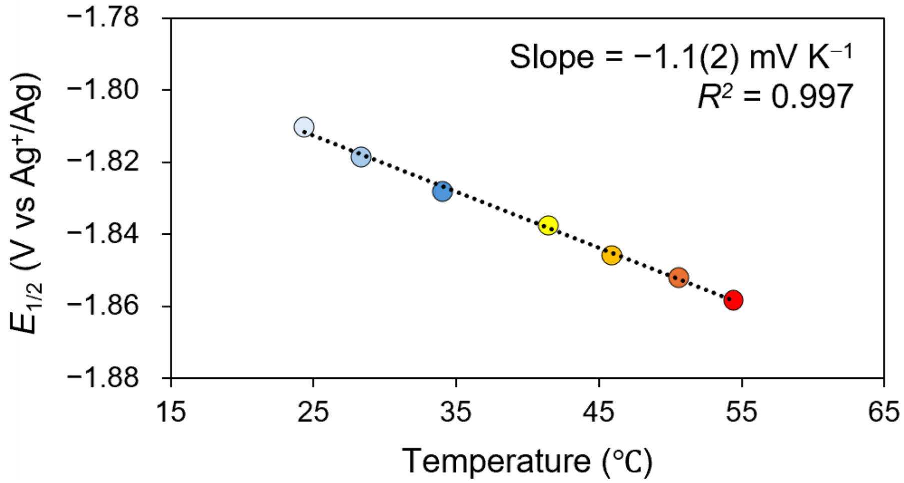

Figure S28. Representative example of the observed temperature dependence of the $E_{1/2}$ values obtained from the corresponding CVs for the 5−/6− redox couple of **PW$_{12}$** as shown in **Figure S25**. The colored circles denote the experimental data, and the black dotted line represents the linear fit to the data. The reported slope (in values of mV K^{-1}, as is the convention in the field) is an average of three independent measurements and the error in the slope corresponds to the standard deviation obtained from these measurements.

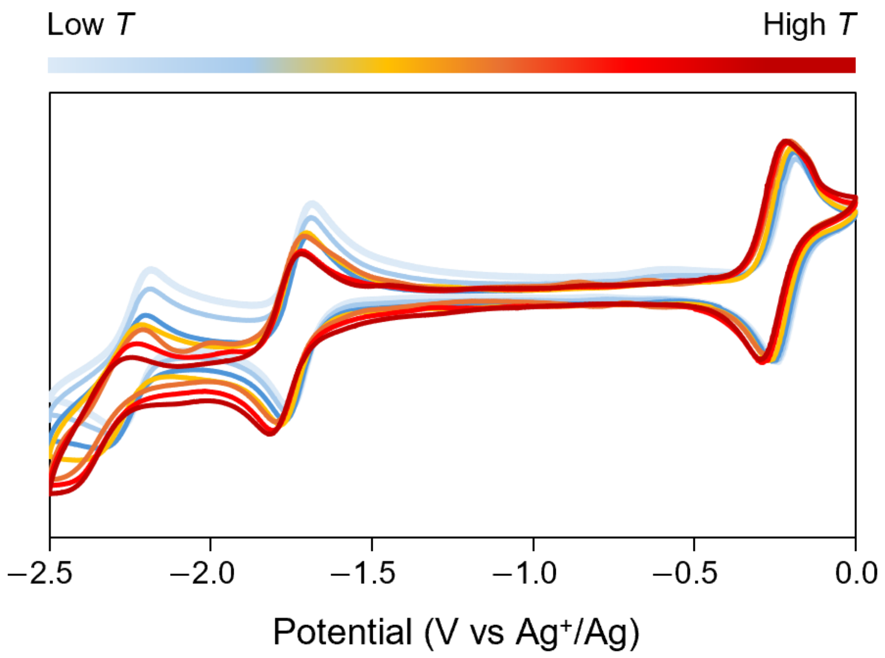

Figure S29. VT-CV measurement of 1 mM solution of **PV$_{out}$W$_{11}$** in acetonitrile with 0.1 M (^{n}Bu$_4$N)(PF$_6$) as the supporting electrolyte, collected at a scan rate of 100 mV s^{-1} using isothermal electrochemical setup from a low temperature of 23.5 °C (blue) to a high temperature of 57.9 °C (red). See the corresponding plots of $E_{1/2}$ vs temperature in **Figures S30** and **S31**.

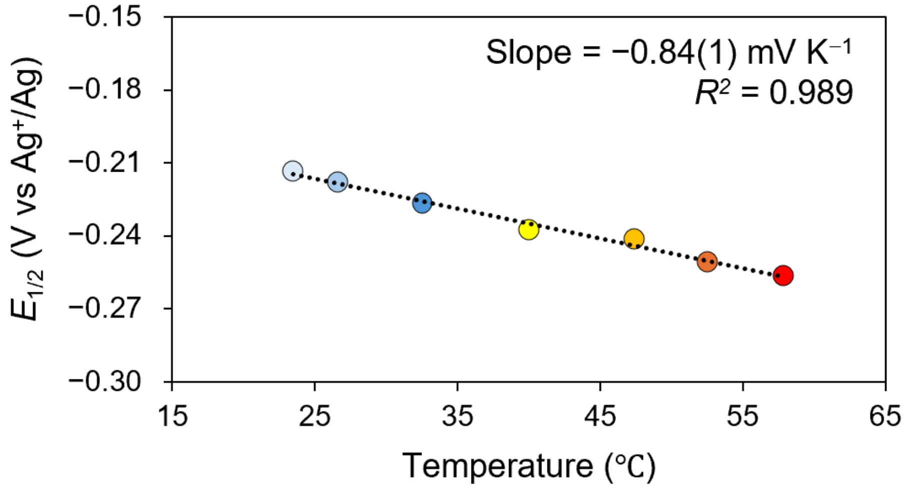

Figure S30. Representative example of the observed temperature dependence of the $E_{1/2}$ values obtained from the corresponding CVs for the 4−/5− redox couple of **PV$_{out}$W$_{11}$** as shown in **Figure S29**. The colored circles denote the experimental data, and the black dotted line represents the linear fit to the data. The reported slope (in values of mV K^{-1}, as is the convention in the field) is an average of three independent measurements and the error in the slope corresponds to the standard deviation obtained from these measurements.

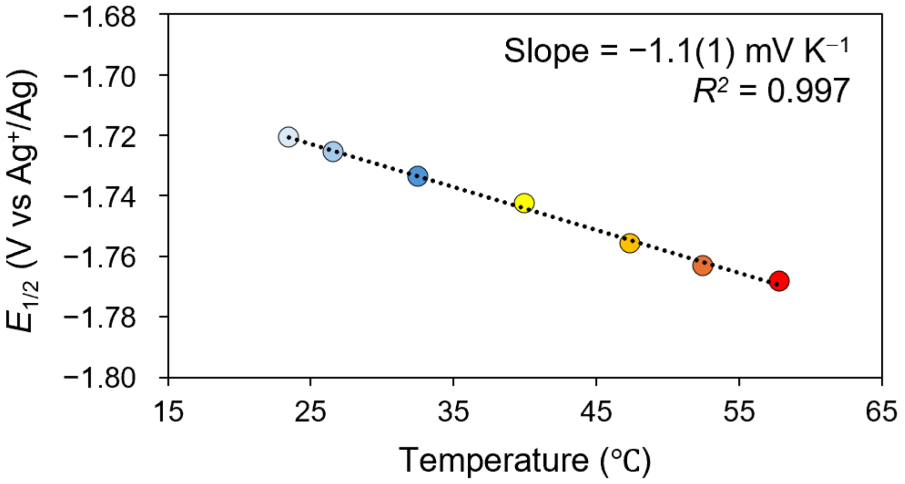

Figure S31. Representative example of the observed temperature dependence of the $E_{1/2}$ values obtained from the corresponding CVs for the 5−/6− redox couple of **PV$_{out}$W$_{11}$** as shown in **Figure S29**. The colored circles denote the experimental data, and the black dotted line represents the linear fit to the data. The reported slope (in values of mV K^{-1}, as is the convention in the field) is an average of three independent measurements and the error in the slope corresponds to the standard deviation obtained from these measurements.

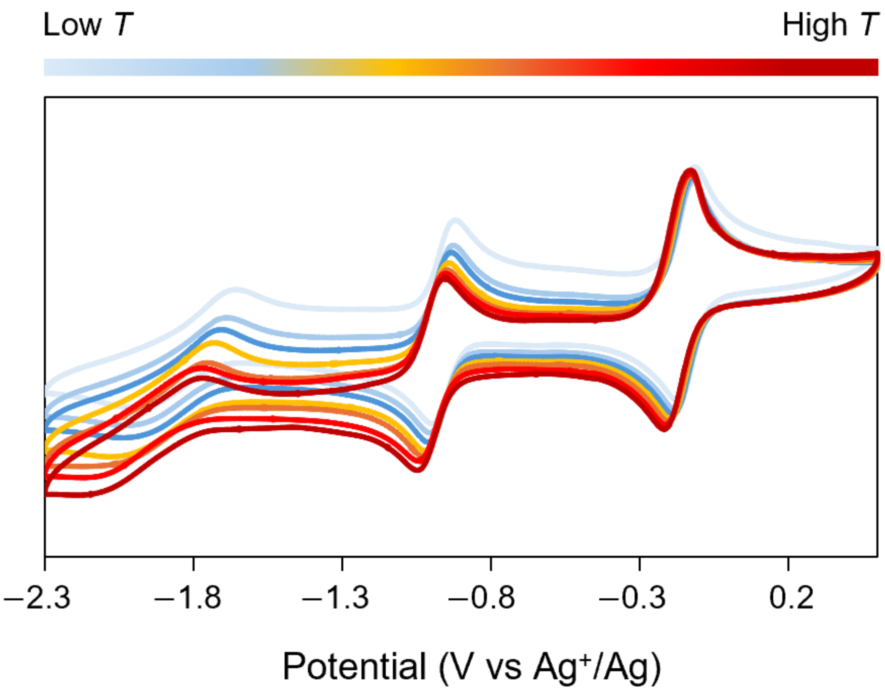

Figure S32. VT-CV measurement of 1 mM solution of **V$_{in}$V$_{out}$W$_{11}$** in acetonitrile with 0.1 M ($^{n}Bu_4N$)(PF_6) as the supporting electrolyte, collected at a scan rate of 100 mV s^{-1} using isothermal electrochemical setup from a low temperature of 21.8 °C (blue) to a high temperature of 52.7 °C (red). See the corresponding plots of $E_{1/2}$ vs temperature in **Figures S33** and **S34**.

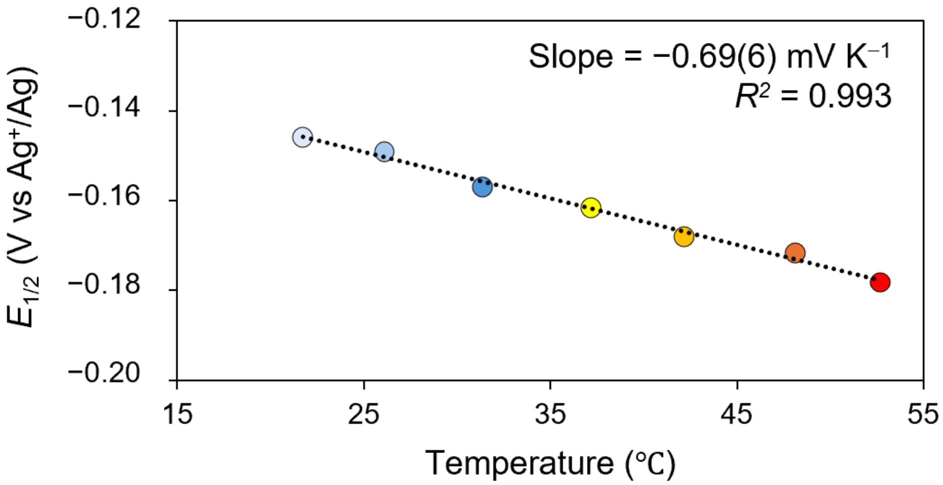

Figure S33. Representative example of the observed temperature dependence of the $E_{1/2}$ values obtained from the corresponding CVs for the 4−/5− redox couple of **$V_{in}V_{out}W_{11}$** as shown in **Figure S32**. The colored circles denote the experimental data, and the black dotted line represents the linear fit to the data. The reported slope (in values of mV K^{-1}, as is the convention in the field) is an average of three independent measurements and the error in the slope corresponds to the standard deviation obtained from these measurements.

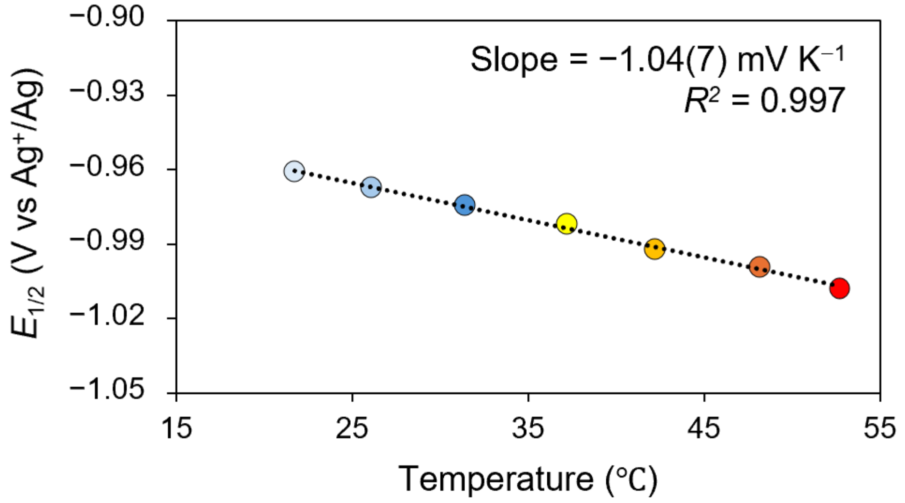

Figure S34. Representative example of the observed temperature dependence of the $E_{1/2}$ values obtained from the corresponding CVs for the 5−/6− redox couple of **$V_{in}V_{out}W_{11}$** as shown in **Figure S32**. The colored circles denote the experimental data, and the black dotted line represents the linear fit to the data. The reported slope (in values of mV K^{-1}, as is the convention in the field) is an average of three independent measurements and the error in the slope corresponds to the standard deviation obtained from these measurements.

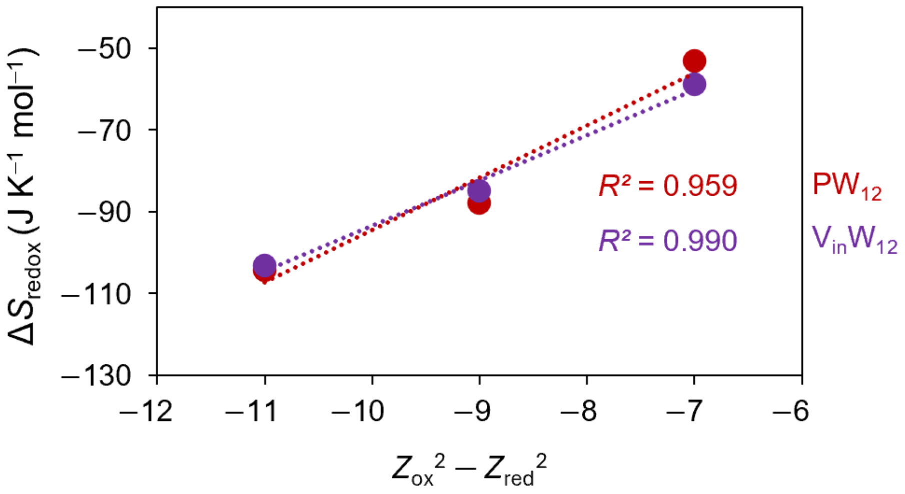

Figure S35. Plot of average redox entropy (ΔS_{redox}) as a function of the dielectric continuum function $Z_{ox}^2 - Z_{red}^2$ for **PW_{12}** and **$V_{in}W_{12}$** as derived from $E_{1/2}$ vs temperature data shown in **Figure S25** and **Figure 4**, respectively, collected in acetonitrile containing 0.1 M (^{n}Bu$_4$N)(PF$_6$) supporting electrolyte using isothermal electrochemical setup. The coefficient of determination (R^2) indicates the goodness-of-fit of the Born expression for the redox entropy for the respective clusters. The colored circles (**PW_{12}**, red; **$V_{in}W_{12}$**, purple) correspond to the experimental data and the colored dotted lines represent the linear fits to the data for the respective clusters.

C. Supplementary Tables

Table S1. Diffusion coefficients for the cathodic wave of one-electron redox couples of $\mathbf{PW_{12}}$, $\mathbf{V_{in}W_{12}}$, $\mathbf{PV_{out}W_{11}}$, and $\mathbf{V_{in}V_{out}W_{11}}$, as calculated from Randles–Ševčík analysis (eqs 1 and 2 in the main text) at 25 °C.[1,2]

POM	Redox couple	Diffusion coefficient, D_0 ($cm^2\ s^{-1}$)		
		Reversible[a]	Irreversible[b]	Average[c]
$\mathbf{PW_{12}}$	3−/4−	9.9(2) × 10^{-6}	1.6(1) × 10^{-5}	1.3(1) × 10^{-5}
	4−/5−	8.9(3) × 10^{-6}	1.4(1) × 10^{-5}	1.2(1) × 10^{-5}
	5−/6−	8.0(3) × 10^{-6}	1.3(1) × 10^{-5}	1.1(1) × 10^{-5}
$\mathbf{V_{in}W_{12}}$	3−/4−	6.2(6) × 10^{-6}	1.0(1) × 10^{-5}	8.1(2) × 10^{-6}
	4−/5−	4.3(1) × 10^{-6}	7.0(1) × 10^{-6}	5.6(1) × 10^{-6}
	5−/6−	5.6(1) × 10^{-6}	9.0(1) × 10^{-6}	7.3(1) × 10^{-6}
$\mathbf{PV_{out}W_{11}}$	4−/5−	6.3(8) × 10^{-6}	1.0(1) × 10^{-5}	8.2(2) × 10^{-6}
	5−/6−	5.1(1) × 10^{-6}	8.2(1) × 10^{-6}	6.7(1) × 10^{-6}
$\mathbf{V_{in}V_{out}W_{11}}$	4−/5−	6.6(1) × 10^{-6}	1.1(1) × 10^{-6}	8.6(1) × 10^{-6}
	5−/6−	6.2(2) × 10^{-6}	1.0(1) × 10^{-5}	8.1(1) × 10^{-6}

[a]The reversible form of the Randles–Ševčík equation (eq 1 in the main text) was used to estimate the reported D_0 values. The errors denote the standard error obtained for the slopes of the linear fits to the $i_{p,c}$ vs $v^{1/2}$ data in the 95% confidence interval of the regression analysis.
[b]The irreversible form of the Randles–Ševčík equation (eq 2 in the main text) was used to estimate the reported D_0 values. The errors denote the standard error obtained for the slopes of the linear fits to the $i_{p,c}$ vs $v^{1/2}$ data in the 95% confidence interval of the regression analysis.
[c]The reported errors are estimated using the average errors of the D_0 values reported for reversible and irreversible diffusion coefficients.

Table S2. Diffusion coefficients for the anodic wave of one-electron redox couples of $\mathbf{PW_{12}}$, $\mathbf{V_{in}W_{12}}$, $\mathbf{PV_{out}W_{11}}$, and $\mathbf{V_{in}V_{out}W_{11}}$, as calculated from Randles–Ševčík analysis (eqs 1 and 2 in the main text) at 25 °C.[1,2]

POM	Redox couple	Diffusion coefficient, D_0 ($cm^2\ s^{-1}$)		
		Reversible[a]	Irreversible[b]	Average[c]
$\mathbf{PW_{12}}$	3−/4−	$1.1(1) \times 10^{-5}$	$1.7(1) \times 10^{-5}$	$1.4(1) \times 10^{-5}$
	4−/5−	$8.8(3) \times 10^{-6}$	$1.4(1) \times 10^{-5}$	$1.2(1) \times 10^{-5}$
	5−/6−	$6.1(2) \times 10^{-6}$	$9.9(2) \times 10^{-6}$	$7.9(2) \times 10^{-6}$
$\mathbf{V_{in}W_{12}}$	3−/4−	$7.6(7) \times 10^{-6}$	$1.2(1) \times 10^{-5}$	$9.9(2) \times 10^{-6}$
	4−/5−	$5.1(2) \times 10^{-6}$	$8.3(2) \times 10^{-6}$	$6.7(2) \times 10^{-6}$
	5−/6−	$5.8(1) \times 10^{-6}$	$9.4(2) \times 10^{-6}$	$7.6(2) \times 10^{-6}$
$\mathbf{PV_{out}W_{11}}$	4−/5−	$6.9(8) \times 10^{-6}$	$1.1(1) \times 10^{-5}$	$9.0(3) \times 10^{-6}$
	5−/6−	$5.8(1) \times 10^{-6}$	$9.4(1) \times 10^{-6}$	$7.6(1) \times 10^{-6}$
$\mathbf{V_{in}V_{out}W_{11}}$	4−/5−	$7.6(2) \times 10^{-6}$	$1.2(1) \times 10^{-5}$	$9.9(2) \times 10^{-6}$
	5−/6−	$6.3(2) \times 10^{-6}$	$1.0(1) \times 10^{-5}$	$8.3(2) \times 10^{-6}$

[a]The reversible form of the Randles–Ševčík equation (eq 1 in the main text) was used to estimate the reported D_0 values. The errors denote the standard error obtained for the slopes of the linear fits to the $i_{p,a}$ vs $v^{1/2}$ data in the 95% confidence interval of the regression analysis.
[b]The irreversible form of the Randles–Ševčík equation (eq 2 in the main text) was used to estimate the reported D_0 values. The errors denote the standard error obtained for the slopes of the linear fits to the $i_{p,a}$ vs $v^{1/2}$ data in the 95% confidence interval of the regression analysis.
[c]The reported errors are estimated using the average errors of the D_0 values reported for reversible and irreversible diffusion coefficients.

Table S3. Radii of gyration for $\mathbf{PW_{12}}$, $\mathbf{V_{in}W_{12}}$, $\mathbf{PV_{out}W_{11}}$, and $\mathbf{V_{in}V_{out}W_{11}}$, as calculated using Multiwfn 3.8(dev) by adopting the crystal structures of the respective Keggin clusters.[3,4]

POM	Radius of gyration (Å)
$\mathbf{PW_{12}}$	3.61
$\mathbf{V_{in}W_{12}}$	3.62
$\mathbf{PV_{out}W_{11}}$	3.60
$\mathbf{V_{in}V_{out}W_{11}}$	3.29

D. References

(1) Randles, J. E. B. A Cathode Ray Polarograph. Part II.—The Current-Voltage Curves. *Trans. Faraday Soc*. **1948**, *44*, 327–338.

(2) Ševčík, A. Oscillographic Polarography with Periodical Triangular Voltage. *Collect. Czech. Chem. Commun*. **1948**, *13*, 349–377.

(3) Lu, T.; Chen, F. Multiwfn: A Multifunctional Wavefunction Analyzer. *J. Comput. Chem*. **2012**, *33* (5), 580–592.

(4) Lu, T. A Comprehensive Electron Wavefunction Analysis Toolbox for Chemists, Multiwfn. *J. Chem. Phys*. **2024**, *161*, No. 082503.
